# The Sv DC Field: Large-Scale and Hierarchical Functions Regulator

**DOI:** 10.34133/research.1476

**Published:** 2026-11-13

**Authors:** Kuiying Yin, Tao Yu, Ming Xiao, Rumei Li, Ying Gao, Shixin Ding, Yimin Zhang

**Affiliations:** ^1^ Link Sense Laboratory, Nanjing Research Institute of Electronic Technology, Nanjing, Jiangsu, China.; ^2^ National Center of Gerontology, Beijing Hospital, Beijing, China.; ^3^ School of Electronic Science and Engineering, Nanjing University, Nanjing, Jiangsu, China.; ^4^ Beijing Institute of Functional Neurosurgery, Department of Neurosurgery, Xuanwu Hospital of Capital Medical University, Beijing, China.; ^5^ Jiangsu Province Key Laboratory of Neurodegeneration, Nanjing Medical University, Nanjing, Jiangsu, China.; ^6^ National Key Laboratory of Radar Detection and Sensing, Nanjing, Jiangsu, China.

## Abstract

Large-scale dynamic reorganization of brain functional networks has been extensively revealed by functional magnetic resonance imaging, yet its direct electrophysiological basis remains unclear. In our prior research on epilepsy, we identified a distinctive electrophysiological phenomenon referred to as the slow-varying direct current (Sv DC, 0.01 to 0.5 Hz) field. This field demonstrates both the stability characteristic of a physical direct current and the slow-varying property of bioelectrical signals, enabling it to reflect the dynamic alterations in relative bioelectric field fluctuations. To further characterize its behavior across different functional states, we recorded intracranial electroencephalography from epilepsy patients implanted with depth electrodes during visual, motor, and language tasks. The Sv DC field exhibited large-scale convergence and suppression, accompanied by information transfer to other frequency bands, during these tasks. In visual functional areas, Sv DC field displayed rhythmic oscillations tightly coupled with gamma activity and increased power, whereas in nonvisual areas, Sv DC field power decreased significantly. During seizures, Sv DC field showed characteristic spatiotemporal spreading dynamics, a phenomenon consistently replicated in macaque monkeys, rats, and mice. These electrophysiological patterns suggest that the Sv DC field exhibits both large-scale cortical functional networks and region-specific modulations, potentially offering a novel understanding on dynamic organization of brain functional networks.

## Introduction

The human brain demonstrates remarkably efficient information-processing capabilities, which are predominantly ascribed to the distinctively layered and modular architecture of the cerebral cortex [1]. Lower-order cortical regions, including the primary visual cortex (V1) and the primary somatosensory/motor cortices (S1/M1), are marked by densely packed subcortical white matter fibers that constitute highly specialized processing units committed to modality-specific sensory or motor functions. Conversely, higher-order brain regions, such as the default mode network, prefrontal cortex, and limbic system, display sparse long-range connectivity. This architecture facilitates cross-modal information integration and abstract cognitive processing, allowing for the emergence of complex mental functions via dynamic network reconfiguration. Evidence of this hierarchical and modular information processing primarily originates from functional magnetic resonance imaging (fMRI) studies [1–4]. Nevertheless, the mechanisms through which the brain attains efficient large-scale functional organization across these hierarchies are still inadequately comprehended, especially owing to the dearth of direct electrophysiological evidence.

Electroencephalography (EEG) offers a direct window into the brain’s electrical activity with millisecond-scale temporal resolution [5]. Conventional EEG recordings employ alternating current-coupled amplifiers with high-pass filters typically set at 0.5 Hz or above to reduce motion and sweat artifacts, which systematically dismisses slow components below this cutoff [6]. Yet brain direct current (DC) potentials—sustained shifts and slow deflections of cerebral potentials superimposed on conventional EEG—have been recognized since the earliest days of EEG [7]. A growing body of evidence has demonstrated that infraslow activity (<0.5 Hz), including DC shifts and slow cortical potentials, is not artifact from eye or head movement or poor contact connection but carries physiologically meaningful information [7–9]. Recent reviews have emphasized that infraslow macroscopic activity involves global coordinated changes of neural activity, neuromodulatory systems, and vascular signals and may operate as a homeostatic mechanism during wakefulness [10–12]. However, for a long time, this field has suffered from naming inconsistencies and ambiguous physical properties. Previous reports on DC shifts during seizures merely described localized manifestations of the slow-varying direct current (Sv DC) in single leads or 2-dimensional cortical planes. We upgrade traditional 2-dimensional signal descriptions to a 3-dimensional macroscopic physical definition of the electric field, explicitly identifying the common essence of various slow-changing electrical activities as the intracranial Sv DC field, resolving a long-standing conceptual divergence in the field [13]. This conceptual framework provides a novel theoretical basis for understanding macroscopic brain electrical regulation.

Notably, the cellular origins of infraslow activity may extend beyond neurons. Emerging evidence indicates that astrocytes, long regarded as passive support elements, contribute to DC shifts, potentially driven by potassium dynamics [14]. Astrocytes exhibit low-frequency membrane potential fluctuations and may play a role in shaping neuronal plasticity and activity [15]. Computational models have shown that the inclusion of astrocytes introduces slow and infraslow oscillations through distinct mechanisms [16]. In vivo studies demonstrate that astrocytic calcium activity precedes spontaneous circuit shifts to slow-oscillation-dominated states [17]. A more recent study further illustrates that astrocytes can establish long-range neural networks that stably connect multiple regions across cerebral hemispheres, with the resulting network patterns showing significant disparities from known neuronal networks [18]. Together, these findings suggest that glial networks may contribute to the generation and modulation of infraslow electrophysiological activity, potentially linking metabolic processes to large-scale brain dynamics.

In the context of epilepsy, ictal DC shifts (infraslow activity) can be recorded in over 90% of patients evaluated with intracranial electrodes [19,20], and infraslow activity exists as a fundamental component of wideband cortical dynamics in focal epilepsy, with evidence of phase-amplitude coupling and functional connectivity in the infraslow band [21,22]. Particularly, ictal DC shifts have been suggested to reflect impaired homeostasis of extracellular potassium by astrocyte dysfunction [20,23]. The Sv DC component (0.01 to 0.5 Hz) that we recently identified accounts for a high-power proportion during seizures, exhibiting spatially dynamic changes correlated with high-frequency activity [13]. The Sv DC field also displays distinct transitions corresponding to task initiation and termination, suggesting a potential role in large-scale brain coordination [13].

Nevertheless, the relationship between Sv DC dynamics and task-specific cortical activation in distinct functional systems (e.g., visual, motor, and language networks) remains unexplored. While astrocytes have been implicated in DC shifts, the temporal relationship between glial activity and Sv DC field dynamics during physiological and pathological states requires further elucidation. It is also unknown whether the Sv DC changes observed in human seizures reflect a species-general phenomenon or are specific to the primate brain.

To address the above issues, the present study intends to characterize the dynamics of the Sv DC field in epilepsy patients under different cognitive task conditions, investigate its underlying principles, and explore its potential relationship with functional cortical activation and energy redistribution. Furthermore, to determine whether the Sv DC dynamics observed during seizures in humans reflect a phylogenetically conserved phenomenon, we examine the Sv DC field in monkey and rodent epilepsy models. In a separate mouse experiment, astrocytic Ca^2+^ signals were recorded alongside conventional EEG. Through these observations, we aim to uncover the electrophysiological basis of how the brain organizes hierarchical networks to support efficient cognitive function in large-scale cortical functional networks.

## Results

### Manifestation of the Sv DC field during seizures

A total of 37 seizures were documented from 12 enrolled patients. The dynamic alterations of Sv DC during the seizure process were analyzed for all patients (Pt. J was excluded from the analysis because of poor ground contact resulting from movement artifacts during the seizure). The findings indicated that, despite the fact that the seizure onset zone can be localized based on conventional frequency bands (0.5 to 140 Hz) using stereo-electroencephalography (SEEG) electrodes, Sv DC components (0.01 to 0.5 Hz) demonstrated a spatial distribution pattern that was markedly distinct from the traditional discharge patterns in the conventional frequency bands during seizures.

For instance, in Pt. P, as depicted in Fig. 1, at the commencement of the seizure, a general decrease in signal amplitude was detected across all electrodes within the conventional frequency bands (Fig. 1A). Conversely, on the Sv DC, electrodes situated within the seizure onset zone (electrodes B and C) promptly demonstrated a distinct spatial dispersion of the Sv DC field. Subsequently, electrodes located in the vicinity but outside the seizure onset zone (electrode A) also manifested a similar dispersion phenomenon. In comparison, electrodes farther from the seizure focus (electrode G) did not exhibit significant dispersion (Fig. 1B). After calculating the Pearson adjacency matrix using a 10-s sliding window for the contacts of electrodes B, A, and G in the Sv DC band, it was found that the correlation between electrodes gradually increased after the onset of a seizure, eventually reaching a state of strong synchronization (Fig. 1C). Moreover, Sv DC changes were primarily characterized by a sustained shift in the DC components, rather than abrupt, instantaneous waveforms. This temporal progression persisted throughout the seizure onset and early propagation phases. These phenomena were consistently observed across diverse patients, suggesting stable dynamic characteristics of Sv DC during seizures. The Sv DC field changes during seizures in different patients have been summarized in Fig. S1, with each patient presenting the EEG of one seizure event. This finding is consistent with our prior research [13], in which we hypothesized that “abnormal alterations in the Sv DC field (such as spreading phenomena) may contribute to the initiation of seizures by shaping the pathological electrical environment.”

**Fig. 1.**
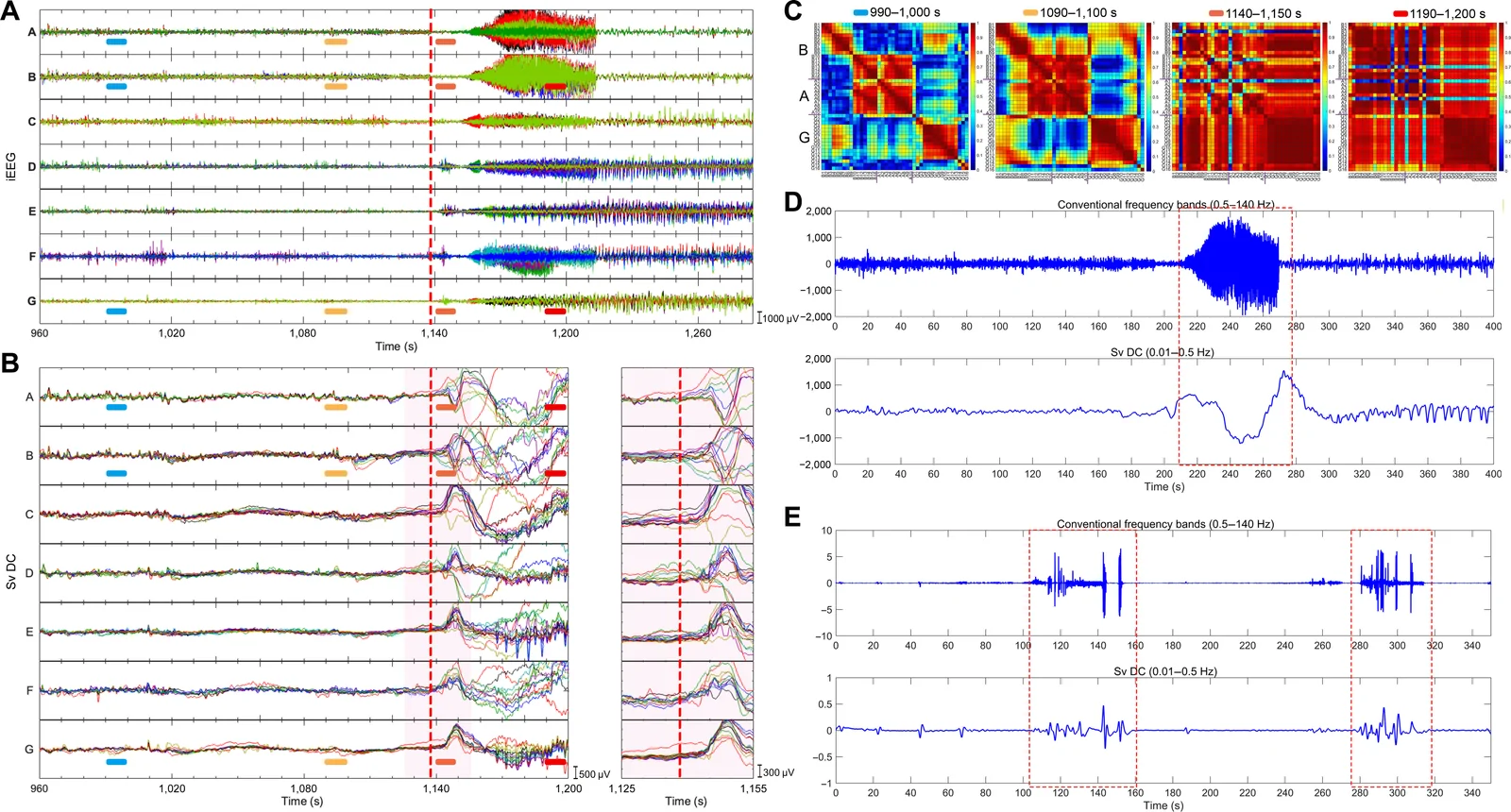
Seizure event in Pt. P. and 1 macaque. (A) Conventional frequency-band activity (0.5 to 140 Hz) across all electrode contacts during a seizure in Pt. P. (B) Left: Slow-varying direct current (Sv DC) signals (0.01 to 0.5 Hz) across all electrode contacts during the same seizure. Right: Enlarged view of the red shaded region shown in the left panel. (C) Pearson adjacency matrices between all contacts of electrodes B, A, and G in Sv DC during different time periods of this seizure. Blue, yellow, orange, and red blocks indicate 4 distinct time windows. The red dashed line marks the seizure onset. (D) Waveforms of single-channel intracranial data from Pt. P during epileptic seizures in the conventional frequency band and at Sv DC. The red box indicates the time interval of the epileptic seizure. (E) Waveforms of single-channel intracranial recording data from 1 macaque.

In addition to human intracranial recordings, this study also collected and analyzed electrophysiological signals from drug-induced seizure models in macaques. Figure 1D and E presents a comparison between conventional frequency-band signals and Sv DC signals acquired from a single channel across the 2 species. Signals recorded from human and macaque during epileptic activity display highly consistent alteration patterns: Both groups exhibit prominent fluctuation characteristics within the ultra-low frequency band, and the amplitude of Sv DC fluctuations during ictal periods is significantly larger than that during interictal periods.

### Manifestation of the Sv DC field in visual function

Six patients with SEEG electrodes implanted in the occipital region demonstrated consistent outcomes during visual tasks. In comparison to the resting state, the Sv DC signals recorded from all intracranial electrodes displayed convergent depression (amplitude reduction accompanied by signal clustering) during visual tasks, with rapid recovery subsequent to task completion (Fig. 2A).

**Fig. 2.**
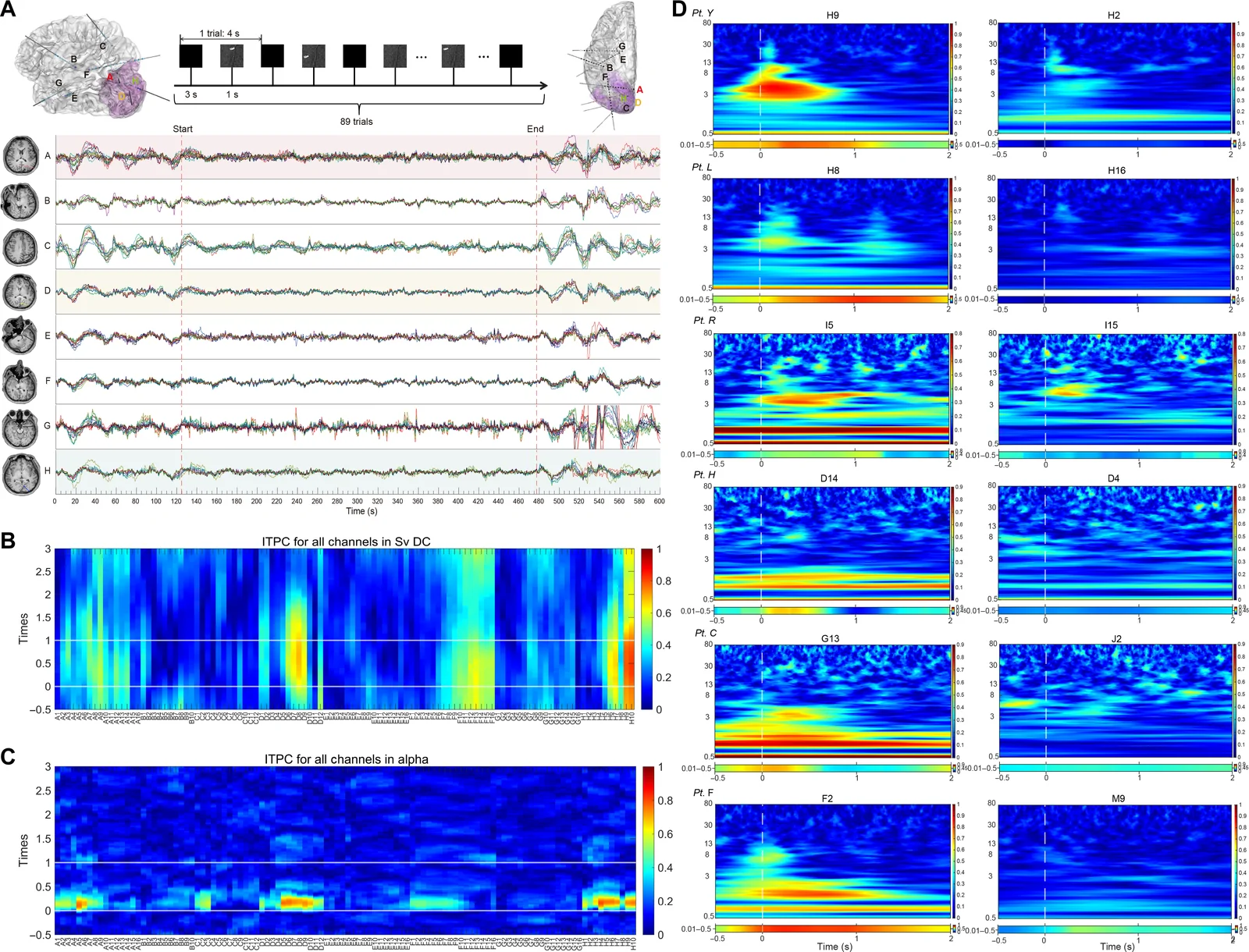
Characteristics of slow-varying direct current (Sv DC) fields during visual tasks. (A) Sv DC schematic diagrams of each electrode in Pt. Y during the performance of visual tasks. The Sv DC signals exhibited convergence and depression (amplitude reduction with signal clustering) during the task, with more pronounced effects observed in the occipital electrodes A, D, and H and the adjacent parietal electrode F. The left panel shows magnetic resonance imaging slices reconstructed along the electrode trajectories. Electrode labels A to H indicate the implantation order. The red dashed lines mark the task onset and offset, and the upper section displays a 3-dimensional (3D) model of the electrode pathways (purple overlay highlights the occipital lobe) and the visual task paradigm. (B) Intertrial phase coherence (ITPC) of Sv DC across all electrodes dynamically varied within the time window of −0.5 to 3 s across 89 trials during the visual task at Pt. Y. The horizontal axis of the figure represents different channels, while the vertical axis corresponds to various time points within the range [−0.5 to 3 s]; the colors in the figure indicate the magnitude of the ITPC values. The task begins at 0 s and ends at 1 s, with the start and end points marked by white lines. (C) ITPC of alpha across all electrodes dynamically varied within the time window of −0.5 to 3 s. (D) ITPC heatmaps of the functional and task low-response associated region regions’ touchpoints of the 6 patients included in this study are presented. The left column shows the touchpoints of the functional regions, while the right column shows those of the task low-response associated region regions. The white dotted line represents the start time of the visual task. Below each heatmap, there is a separate line indicating the Sv DC frequency band, while above it is the 0.5 to 80Hz frequency band.

Taking patient Pt. Y as an example, we used intertrial phase coherence (ITPC) to evaluate neural activity at various electrode sites at different time points during the task execution period in the Sv DC and alpha frequency bands (Fig. 2B and C). In the Sv DC band, the parietal electrode contacts A1 to A14, D5 to D9, and H5 to H10, as well as the adjacent occipital electrode contacts F6 to F16, exhibited task-dependent ITPC enhancement, indicating that the brain regions corresponding to these electrodes were deeply involved in the visual task processing. The ITPC enhancement in the D5 to D9 and H5 to H10 electrode channels consistently persisted until approximately 1.5 s (500 ms after task completion). It is also worth noting that the timing of activation at electrode channels in various vision-related brain regions under Sv DC conditions varies: ITPC enhancement at F6 to F16 occurred even before the task began, with the enhancement occurring between [−0.5 and 1 s], whereas electrodes D5 to D9 showed marked enhancement within the [0 to 1.5 s] range. In the alpha band, the F1 to F9 electrode contacts exhibited task-driven phase synchronization; some channels on the B and C electrodes—excluding those in the visual cortex—also showed enhancement, which may correspond to higher-level cognitive and other processing mechanisms. Compared to Sv DC, the enhancement in the alpha band lasted for a shorter duration, primarily concentrated within the first 300 ms after the task began, and had largely disappeared after 300 ms.

In the present study, 6 enrolled patients successfully completed the visual paradigm experiment, from which electrode contacts located in visual functional areas and nonvisual functional areas were separately extracted. ITPC was then calculated, and the corresponding topographic heatmaps were constructed. By comparing the time–frequency dynamics of task-evoked neural activity of electrode contacts in different functional regions after task onset, this study aimed to investigate whether electrode contacts localized to visual functional areas exhibited differentiated task-related neural activity. The experimental results show that after the onset of the visual task, electrode contacts within visual functional areas presented more prominent task-related changes in both waveform characteristics and intertrial phase consistency. By contrast, electrode contacts in nonvisual functional areas did not show similar region-specific response patterns (Fig. 2D).

### Manifestation of the Sv DC field in motor and language functions

Analogous to the visual task, all electrodes manifested convergence and depression (characterized by amplitude reduction accompanied by signal clustering) of the Sv DC field during the performance of simple motor actions (Task 1: fanning), fine motor tasks (Task 2: transporting beans with a spoon), and complex gesture tasks (Task 3: finger counting). The extent of convergence was correlated with task complexity; more precise movements gave rise to stronger convergence, with Task 2 and Task 3 exhibiting greater convergence than Task 1 (Fig. 3A). Figure 3B presents the outcomes of intraoperative awake language testing in Patient J utilizing a 32-contact cortical electrode array. During language tasks, all electrodes demonstrated convergence and depression of the Sv DC fields, which promptly reverted to baseline dispersion upon task completion. Notably, the convergence and depression were more prominent during the repetition tasks compared to the picture-naming tasks. Additionally, Fig. S2 depicts consistent task-related convergence and depression of the Sv DC field in 4 supplementary patients (2 language tasks, 1 motor task, and 1 visual task), further corroborating the robustness of this phenomenon across individuals.

**Fig. 3.**
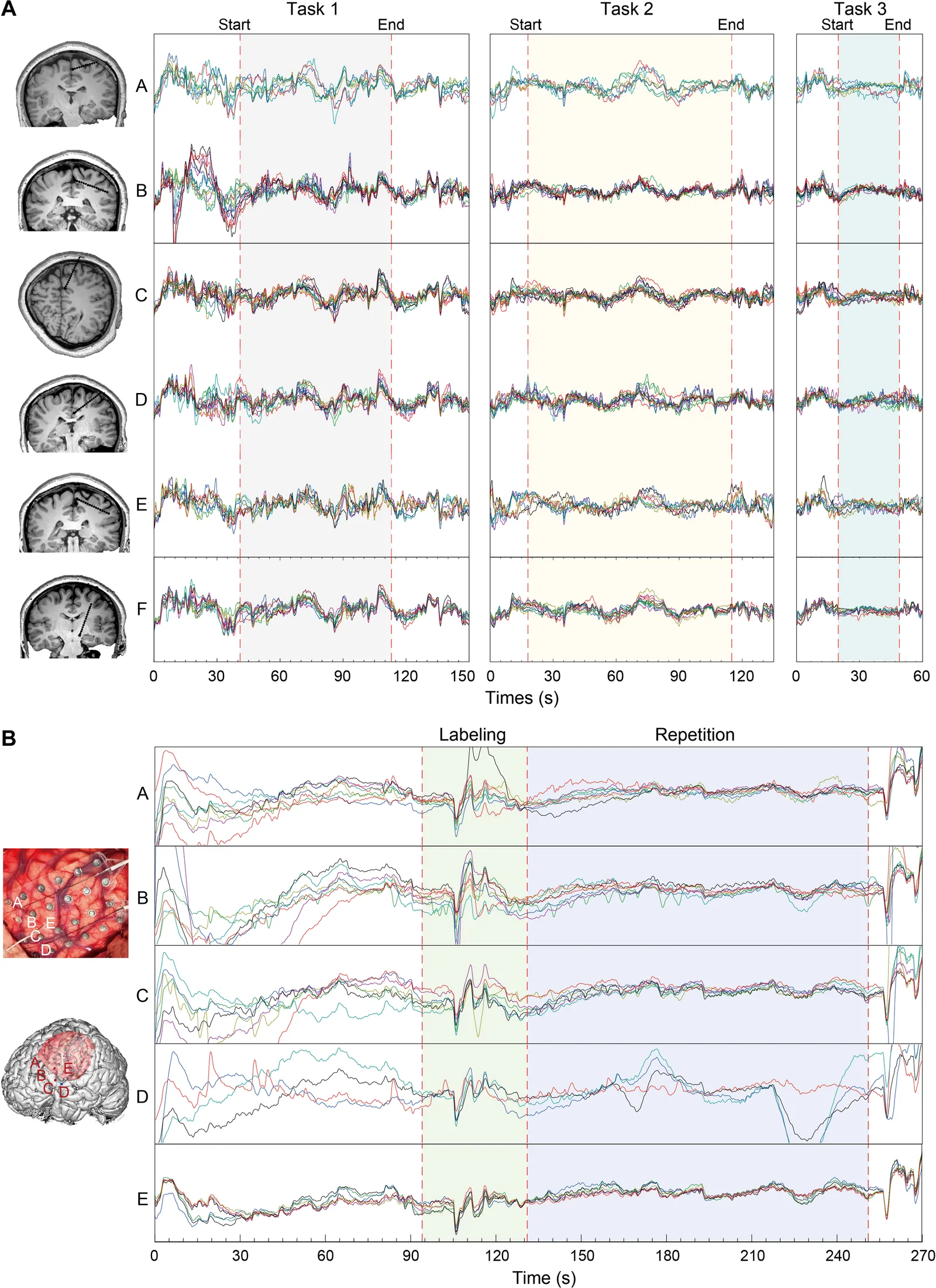
Characteristics of the slow-varying direct current (Sv DC) fields during motor and language tasks. (A) Sv DC changes during different hand movement tasks. The Sv DC traces recorded from all electrodes in Patient W during hand motor tasks demonstrated convergence and depression (amplitude reduction with signal clustering). Three tasks were performed: Task 1 (fanning motion), Task 2 (bean transfer with a spoon), and Task 3 (finger counting gestures). For each task, the red dashed lines indicate the onset and offset time points. The left panel shows the MRI slices reconstructed along the electrode trajectories (A to F: electrode numbering). (B) Changes in the Sv DC fields during awake language tasks in Patient J. The left panel shows the placement of the cortical electrodes (A to D) and 1 depth electrode (E) during surgery. The top-left panel shows an intraoperative photograph of the surgical field, while the bottom-left panel shows the relative position of the surgical field on the patient’s meninges. The right panel presents Sv DC recordings taken during the picture naming and repetition tasks, showing characteristic signal convergence and depression (amplitude reduction) during task performance, which subsequently returned to baseline.

### Spatiotemporal characteristics of Sv DC fields

We conducted an analysis of the waveform and power variations across the electrodes in Pt.Y during visual tasks. Initially, we replicated the event-related potential (ERP) waveforms that were comparable to those of scalp recordings obtained via intracranial electrodes. We observed highly synchronized physiological waves at 100, 150, and 350 ms (Fig. 4A). Based on these specific time points, we compared the Sv DC power changes across frequency bands in the functional area (H9), the association cortex (C10), and the task low-response associated region area (G15) (Fig. 4B). We divided the first 1 s after task onset into 10 consecutive windows with a step size of 100 ms, calculated the log-transformed mean square power for each window, and used a 500-s isochronous resting period prior to the task as the paired baseline. We then performed trend-adjusted regression on the window-by-window power differences (task minus rest) for each trial, using the regression intercept as the average effect within each window, and conducted 2-tailed 1-sided *t* tests on the channels of interest. The results showed that a relatively pronounced increase in Sv DC power was observed at the H9 electrode only during the initial phase of the task (0 to 100 ms) (*P*_fdr = 0.034, |*d*| = 0.27, *β* = 1.55, confidence interval = [0.33, 2.76]), and during the task, the power gradually shifted from being stronger than the resting baseline to weaker than the resting baseline. The difference curve (autoregressive *β* value) at H9 showed a downward trend over time, with the mean difference between the task and resting states approaching 0 at 500 to 600 ms. For the task low-response associated region electrodes C10 and G15, lower Sv DC power levels were observed during the task compared to the resting baseline, and the trend in power differences over time during the task was not pronounced. In fact, except for H9 and F11, which showed an upward trend in power during certain time windows of the task (statistically insignificant for the F11 electrode), the remaining task low-response associated region electrode contacts (B10, C10, E10, and G15) all exhibited a significant decrease in DC power or a mean autoregressive intercept β below the baseline of 0 during task execution (Fig. S3).

**Fig. 4.**
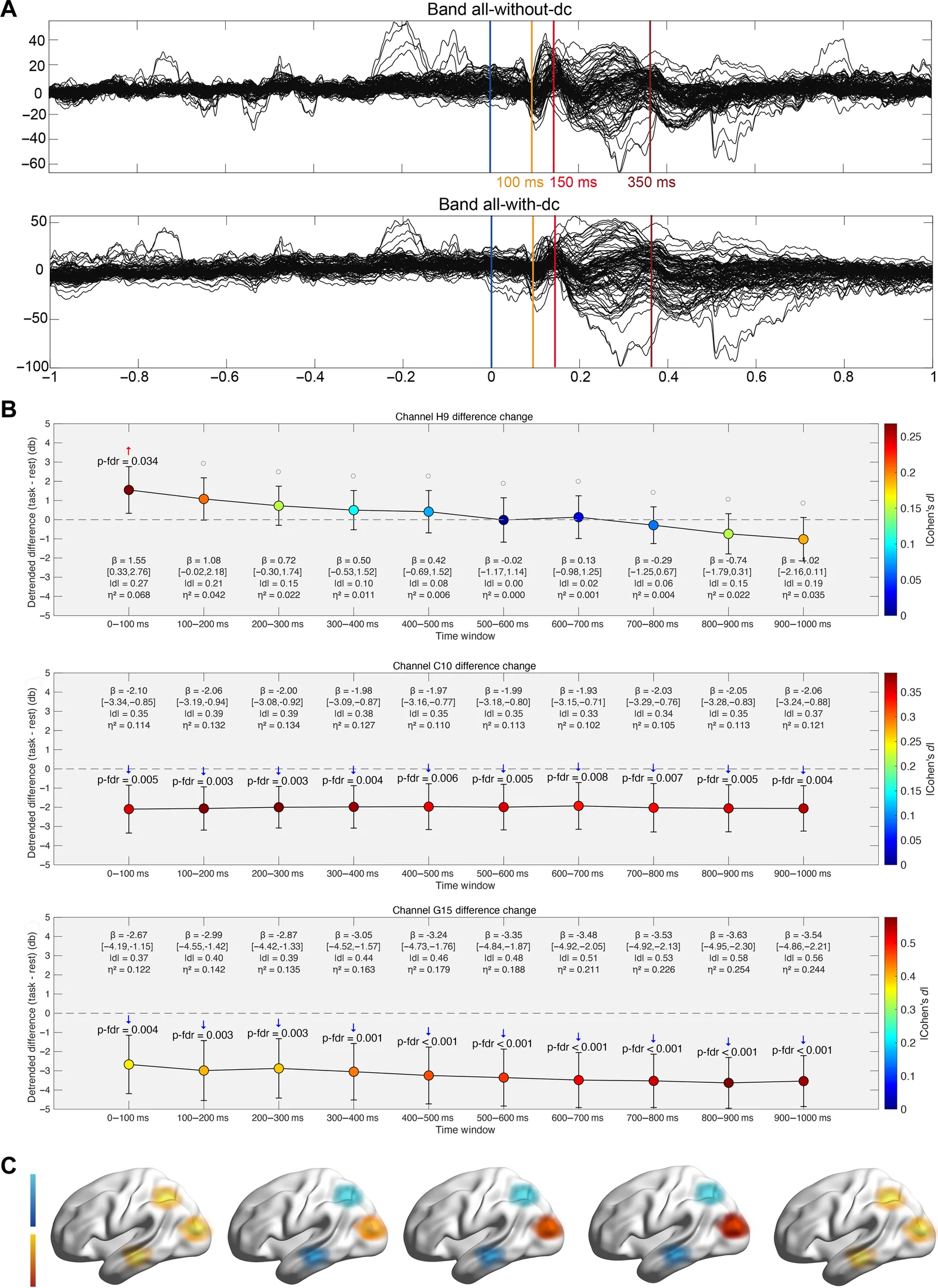
Event-related potential (ERP) reproduction and power changes during visual tasks. (A) Average ERP waveforms from Patient Y, showing both slow-varying direct current (Sv DC)-removed and Sv DC-included signals across all electrodes during task performance. The intracranial recordings revealed significant physiological waves at 100, 150, and 350 ms. (B) Dynamic changes in the power difference between task and resting state for Patient Y, after autoregressive detrending, over the 0- to 1,000-ms interval. The black error bars represent the 95% confidence interval (CI) for the detrended intercept (*β*_0_) in each time window, calculated from the regression standard error based on the t-distribution, reflecting the precision of the effect size estimate. Colored scatter points (circles) represent the mean *β*_0_ values; the shade of color maps the magnitude of the effect size |Cohen’s *d*|, with redder colors indicating a larger effect. Arrow markers indicate that, following false discovery rate correction of the *P* values from 2-tailed 1-sided *t* tests, the power in that time window is significantly different from the resting-state baseline. The text labels, in order, are the *β* value (mean) for that window, the upper and lower limits of the 95% CI, |Cohen’s *d*|, and partial Eta-squared. (C) Meningeal projections of 3 representative electrodes (H9, C10, and G15) with color-coded power changes at key time points (0, 100, 150, 350, and 500 ms). Yellow represents resting-state activity, with progressive red-shifts marking higher power levels, while blue-shifts indicate lower power relative to the baseline at each time point.

Furthermore, the meningeal projections of 3 representative contacts (H9, C10, and G15) were visualized through color coding to illustrate their power changes (Fig. 4C). The complete power dynamics over the entire task period are depicted in Fig. S5. Remarkably, multiple task low-response associated region electrodes (B, C, E, F, and G) demonstrated a significant reduction in DC power during tasks (decrease rate: 0.59 ± 0.30), without corresponding increases in other frequency bands. Consequently, it is hypothesized that this portion of DC power may have been reallocated to functional areas.

To validate this hypothesis, an analysis was conducted on the correlation between the DC power change (the percentage change in Sv DC power values within the 0- to 300-ms window relative to the 300 ms prior to task onset) of each contact and the transfer entropy (TE) from its Sv DC to other frequency bands (0.5 to 249 Hz). The findings indicated no significant correlation between the reduction in DC power and the TE of each contact itself (Fig. S4), lending support to the possibility of cross-regional power allocation. This passive response pattern implies that DC fields might coordinate distributed brain regions by sustaining large-scale electric field homeostasis without depending on local power consumption.

### Local oscillations of Sv DC fields

Beyond comparing the overall Sv DC changes across diverse electrodes, we conducted a more in-depth examination of the local Sv DC activity at individual electrodes. During visual tasks, specific electrodes within the visual cortical regions (e.g., A, F, and H) exhibited distinct rhythmic oscillations that were temporally synchronized with image presentation (Fig. 5A and B). The result graph of another patient who underwent the identical visual paradigm is presented in Fig. S5. We selected representative contacts from each electrode to illustrate their task-related electrical activity patterns.

**Fig. 5.**
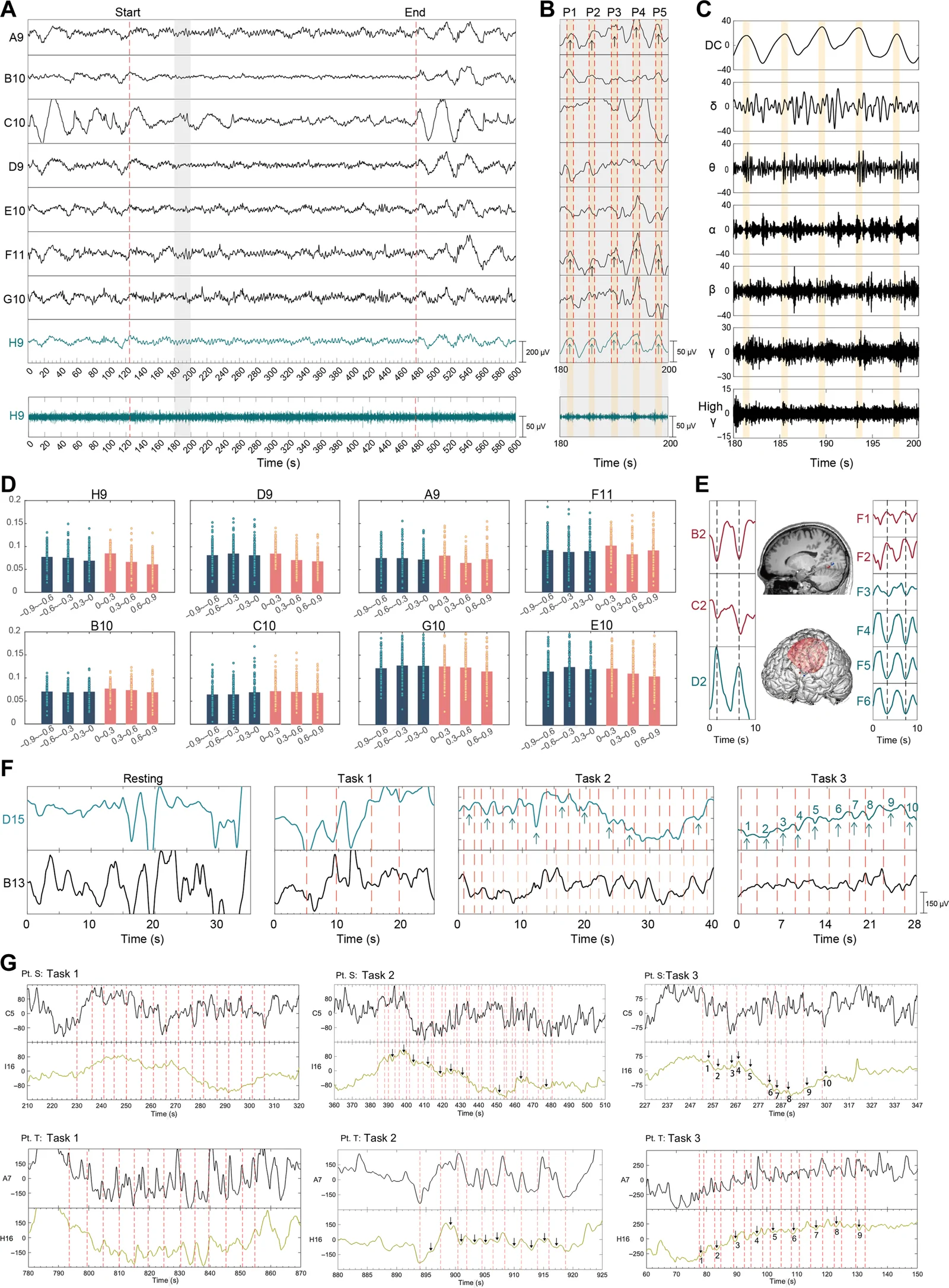
Local oscillations and polarity reversals of the slow-varying direct current (Sv DC) during tasks. (A) Sv DC recordings from representative contacts across electrodes in Patient Y (visual task), along with gamma activity from contact H9. A9: left lateral occipital cortex; B10: left parietal lobe; C10: left parietal lobe; D9: left occipital lobe; E10: left temporal lobe; F11: left parietal lobe; G10: left temporal lobe; H9: left occipital lobe. (B) Detailed Sv DC oscillations during stimulus presentation (corresponding to the shaded period in [A]). The yellow shaded area indicates the 1-s image presentation window, with arrows marking the task-locked waveforms synchronized to the stimulus onset. (C) Multifrequency band activity from contact H9 during the time window shown in (B). (D) Transfer entropy from DC to other frequency bands (0.5 to 249 Hz) for representative contacts in Patient Y, calculated from 0.9 s before to 0.9 s after task onset (300-ms window length). The height of the bar chart in the figure represents the mean transmission entropy across the 89 trials within the current time window, and the scatter points represent the results of the 89 calculations. (E) Patient F and Patient J showed an inversion effect on the transsulcal electrodes (F1, F2, and F3 to 6 (intracranial electrodes) and B3, C3, and D2 (intraoperative cortical electrodes) during the visual and language tasks, respectively. (F) Sv DC activity in Patient W (hand movement tasks) recorded from the motor functional area (D15) and nonfunctional area (B13: left parietal lobe). From left to right: Resting state, Task 1 (fanning), Task 2 (bean transport with spoon), and Task 3 (finger counting). In Task 1, each dashed line marks 10 complete fanning cycles (1 up-down movement equals 1 cycle), reflecting the rapid movement frequency. For Task 2, the first dashed line indicates when the spoon was loaded with beans and movement began, while the second line indicates when the beans were unloaded and the return movement started. During each bean transfer, clear task-related positive waves were observed in the functional area (D15) but not in the nonfunctional area. In Task 3, each number gesture (finger count) is represented between 2 dashed lines. Distinct task-modulated waveforms were consistently detected in D15 during these gestures. (G) Sv DC waveforms from functional and task low-response associated region regions in Patient S (C5: nonfunctional area, right parietal lobe; I16: motor functional area, right precentral gyrus) and Patient T (A7: left frontal lobe, H16: motor functional area, left precentral gyrus) during the same hand movement task.

Furthermore, the contact within the primary visual cortex (V2 area, H9) registered notably heightened gamma activity during the tasks, which also exhibited time-locked synchronization with Sv DC oscillations. Taking contact H9 as an instance, dynamic alterations in electrical activity across diverse frequency bands were distinctly observed during task execution (Fig. 5C).

By calculating the TE from the Sv DC to other frequency bands (0.5 to 249 Hz) at representative contacts and evaluating the results using a permutation repeated-measures analysis of variance (ANOVA), we found significant differences in TE among all electrodes—except for electrode C10—within the [−0.9, 0.9 s] interval. A post hoc paired permutation test was used to further examine differences between pairs of time windows, revealing that the most pronounced changes in TE occurred within the first 300 ms after task onset (Fig. 5D). The detailed results are presented in Tables S1 to S8 in the Supplementary Materials. Furthermore, the time windows showing significant differences were primarily concentrated within the period following task onset. There were no significant differences in TE across the pretask time windows (–0.9 to –0.6, –0.6 to –0.3, and –0.3 to 0); differences were observed only between the posttask and pretask time windows or between different posttask time windows (Tables S2 to S8). More specifically, the TE of H9 and F11 during the 0- to 0.3-s interval showed a significant increase compared to other posttask intervals (0.3 to 0.6, 0.6 to 0.9) and the pretask resting phase (–0.6 to –0.3, –0.3 to 0) (Tables S2 and S5). The TE of electrode A9 during the 0- to 0.3-s interval showed significant differences only between the 2 adjacent time windows (Table S4). The TE of B10 (Table S6) in the nonvisual task area during the 0- to 0.3-s interval was also significantly higher than during other time periods. Changes at electrode D9 were notably smaller in the (0.3 to 0.6, 0.6 to 0.9) time windows. Electrode E10 (Table S8) in the nonvisual task area showed a similar pattern to D9, with relatively smaller values in the posttask (0.3 to 0.6, 0.6 to 0.9) time windows. Differences between time windows at electrode G10 (Table S7) were small, with lower values observed in the 0.6 to 0.9 range.

In addition, daily-routine data recorded before the task (Participant Y, 100 s after recording onset until just before the first trial) were used as the physiological baseline. These data were extracted with a 300-ms sliding window (150-ms step), and TE from Sv DC to other frequency bands was calculated with the same parameters as during the task. Task-related TE and baseline TE were compared using the Mann–Whitney U test (false discovery rate [FDR]-corrected), and median differences were recorded. Only channels and time periods with significant differences were retained, yielding the median change rates (Table S9) from daily state to task ([TE_task − TE_daily] / TE_daily). Compared to the daily state, the changes in TE during the task were consistently enhanced. The time period with the highest percentage increase across all channels occurred within the first 300 ms after task onset, and the greatest change was observed at the H9 electrode located in the visual region of the occipital lobe. Furthermore, throughout the entire task period—even during the time interval preceding the appearance of the task-guiding image (–0.9 to 0)—there were differences in TE compared to the daily state, suggesting that the enhancement in TE from Sv DC to high frequencies persisted throughout the entire task period, whether during the preparation phase before each trial or after task initiation. Taking channels H9 and F11 as examples, Fig. S6 shows that the distribution of TE during task execution (0 to 0.3 s) is more concentrated and stable.

Notably, task-related Sv DC oscillations demonstrated opposite polarities across diverse contacts on certain functional electrodes (Fig. 5E). For instance, during the language task of Patient J, cortical contacts B3 and C2 presented synchronous oscillations, while D2 displayed reversed polarity. This phase reversal was consistent with both the electrode arrangement and sulci anatomy, indicating anatomical influences. A comparable pattern emerged during the visual task of Patient F, where contacts F1/F2 oscillated in an inverse manner to F4 to F6, and magnetic resonance imaging (MRI) coregistration indicated that these contacts were distributed on opposite banks of a cortical gyrus.

We also conducted an analysis of the data obtained from Patient W during the hand movement tasks. The outcomes indicated that the contacts within the motor areas (e.g., D15) likewise demonstrated rhythmic Sv DC oscillations during task execution, and the prominence of these oscillations was closely associated with task complexity. The most pronounced oscillations were observed during complex gesture tasks (Task 3). In the case of moderately difficult tasks (Task 2), the oscillations were more apparent in the early trials but declined as task proficiency improved. Conversely, simple repetitive movements (Task 1) led to almost negligible oscillations (Fig. 5F). In 2 additional patients who completed the identical hand movement task, we observed a pattern that was consistent with that documented in Patient W (Fig. 5G). These findings imply that Sv DC activity not only reflects the task responses in functional areas but may also be correlated with task complexity and cognitive demand.

### Sv DC fields during language tasks and their correspondence with fMRI findings

In order to investigate the spatiotemporal dynamics of language-related Sv DC oscillations and their relationship with fMRI blood oxygenation level-dependent (BOLD) activation, we recorded Sv DC activity in Patient A during language-related tasks. During the picture-naming task (Task 1), distinct Sv DC field oscillations were observed at contact B14 (located in Wernicke’s area) and contact H10 (located in Broca’s area), with the oscillations at B14 appearing to precede those at H10. Similar oscillatory patterns were also observed at contacts C10 and H10 during the question-answering task (Task 2). In the specific trials presented, a temporal delay of 833 ± 215 ms was noted between the onset of oscillations in Wernicke’s area (C10) and Broca’s area (H10) (Fig. 6A).

**Fig. 6.**
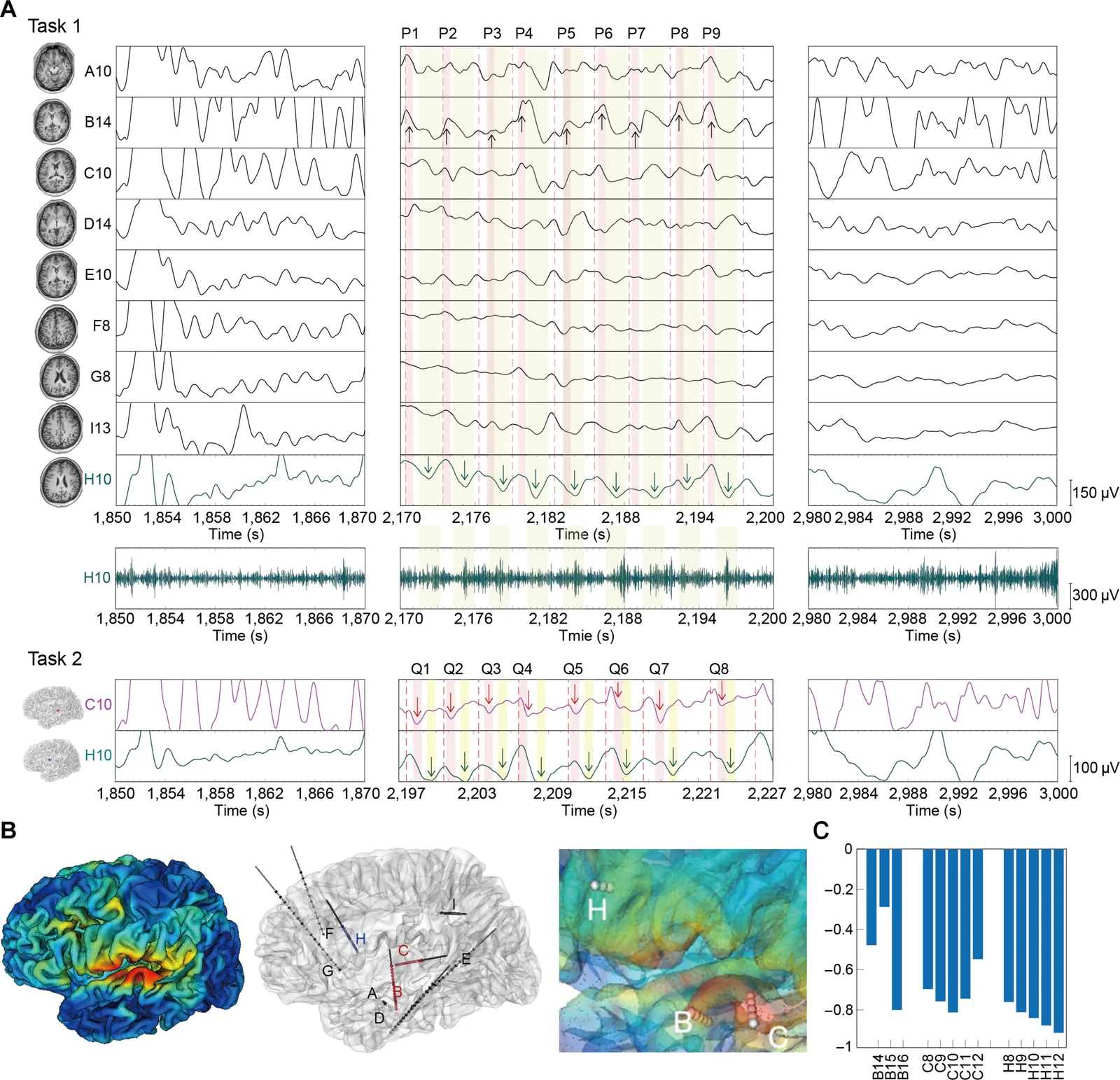
Functional magnetic resonance imaging and slow-varying direct current (Sv DC) manifestations during language tasks. (A) Sv DC recordings from representative electrode contacts during Task 1 (picture naming) and Task 2 (question answering) in this patient, including gamma-band activity from contact H10 in Task 1. The left panels show the pretask baseline Sv DC, the middle panels display the task period activity, and the right panels present the posttask recordings. In Task 1, each dashed line indicates the image presentation onset. Contact B14 consistently showed negative task-related waveforms (black arrows) following image presentation, whereas H10 subsequently displayed clear naming-related waveforms (green arrows). Red shading marks the period during which the patient viewed and comprehended images, while yellow shading indicates speech onset during naming. For Task 2, contacts C10 and H10 presented the most prominent task-modulated Sv DC waveforms. Each dashed line represents the question onset. Red shading (the question comprehension period) corresponded with positive deflections at C10 (red arrows), while yellow shading (answer initiation) was associated with marked activity at H10 (green arrows). The time delay between comprehension and response, as measured by the red and yellow shading intervals, ranged from 392 to 1,044 ms (mean 833 ± 215 ms). The left-side images show the MRI slices reconstructed along electrode trajectories and 3-dimensional 3D meningeal projections, illustrating the spatial locations of the corresponding electrode contacts. (B) Left: Blood oxygenation level-dependent activation map from Patient A’s functional magnetic resonance imaging during auditory comprehension tasks. Middle: Reconstructed electrode trajectories. Right: Relevant electrode contacts in functionally activated areas. (C) Relative change in power of the electrode contacts (B14-16, C8-12, and H8-12) in the question-answering (Task 2) task state in the activated brain region of this patient. A10: left middle temporal gyrus; B14: left superior temporal gyrus; C10: posterior left superior temporal gyrus (Wernicke’s area); D14: left middle temporal gyrus; E10: left temporoparieto-occipital junction (TPO region); F8: left frontal lobe; G8: left frontal lobe; H10: left inferior frontal gyrus (Broca’s area); I13: left parietal lobe.

Additionally, an analysis of the patient’s language fMRI results revealed predominant activation in Wernicke’s area during the auditory comprehension task (Fig. 6B). By superimposing the reconstructed electrode trajectories onto the language fMRI activation map, we observed a spatial overlap between the electrode contacts exhibiting task-related electrophysiological oscillations and the activated brain regions identified via fMRI (Fig. 6B).

Finally, we calculated the relative changes in spectral power ([task-state power − resting-state power]/resting-state power) for the electrode contacts within these functional areas (B14 to 16, C8 to 12, and H8 to 12). All these contacts exhibited a marked increase in spectral power during the tasks (Fig. 6C), a finding that qualitatively aligns with the increased oxygen consumption observed in fMRI during task states compared to resting states. Although the tasks employed during SEEG recording were not identical to the auditory comprehension fMRI paradigm, as an exploratory observation, these Sv DC findings provide potential electrophysiological evidence supporting BOLD signal enhancement.

### Temporal correlation between astrocytic Ca^2+^ rises and Sv DC field changes during seizure onset in a rodent model

We determined whether the electrophysiological phenomenon of the Sv DC field observed in clinical epilepsy patients could also be replicated in epilepsy models in rodents. The onset time point of seizure was determined by the EEG signals (EEG-T0), which is characterized by an abrupt and distinct deviation from the interictal baseline activity with high amplitude. Notably, during the onset phase, a remarkable emergence of the Sv DC field increases was documented in mice (Fig. 7A and B).

**Fig. 7.**
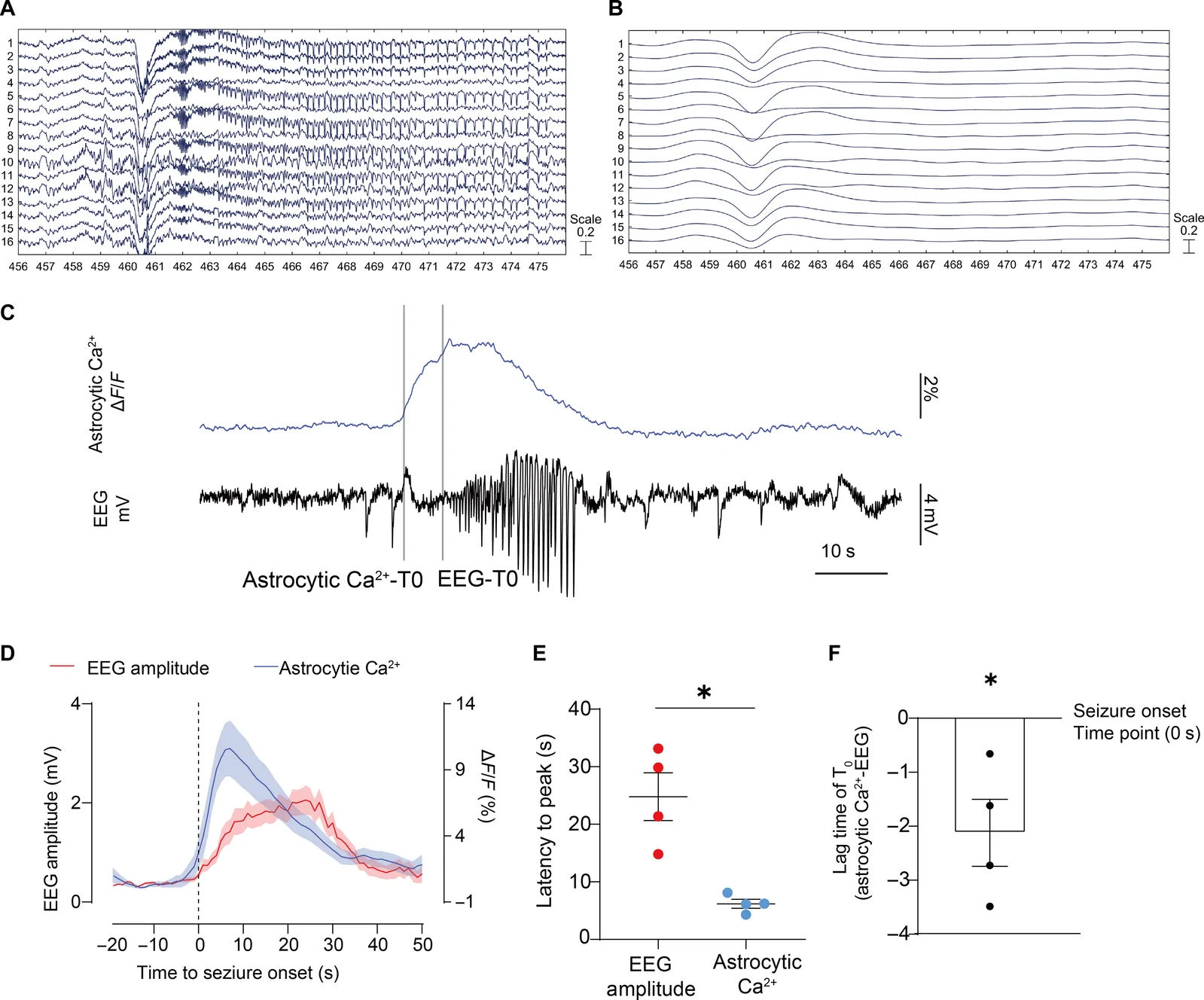
Spatiotemporal dynamics and coupling of low-frequency oscillations and astrocytic Ca^2+^ signals during epileptic seizure onset. (A and B) Hippocampal field-potential recordings and the corresponding 0.01- to 0.5-Hz slow-varying direct current components during chemically induced seizures in mice. The raw acquisition files retained the low-frequency components used for this analysis. These recordings were obtained in a separate experiment from the conventional electroencephalography (EEG) and astrocytic Ca^2+^ photometry shown in Fig. 7C to F. (C) Representative traces of dural recordings of hippocampal astrocytic Ca^2+^ signaling and EEG signals during seizure onset in mice. Scale bar, 10 s for time, 2% for Ca^2+^ signals (Δ*F*/*F*), 4 mV for EEG amplitude. (D) Summarization of in-total 19 seizure onsets from 4 mice. The averaged EEG amplitude (mV) and astrocytic Ca^2+^ signals (Δ*F*/*F*, %) during the time window from 20s prior to the seizure onset till 50s post onset. EEG amplitude was averaged by 1-min epoch. (E) Comparisons of the latency to peak value of EEG amplitude and astrocytic Ca^2+^ signaling. (*n* = 4, paired *t* test, *P < 0.05). (F) The time lag of astrocytic Ca^2+^ elevation time point (astrocytic Ca^2+^-T0) versus the onset time point (EEG-T0). It was compared with the constant 0 sec of seizure onset time point (*n* = 4, 1-sample *t* test, *P < 0.05). Data are presented as mean ± SEM.

In a separate mouse experiment, conventional EEG and hippocampal astrocytic Ca^2+^ signals were recorded during chemogenetically induced seizures. The seizure onset was defined from the conventional EEG trace (EEG-T0), and the astrocytic Ca^2+^ onset was defined when Δ*F*/*F* exceeded the pre-event baseline threshold (mean + 3 SD) (Fig. 7C and D). Astrocytic Ca^2+^ signals occurred significantly earlier than the peak of EEG amplitude at the individual animal level (Fig. 7E). The lag time between astrocytic Ca^2+^-T0 and EEG-T0 was −2.027 ± 1.287 s (Fig. 7F). These results indicate an exploratory temporal association with conventional EEG.

Moreover, a TRPA1 inhibitor, HC-030031, was utilized to impede calcium influx in astrocytes. Electrocorticogram (ECoG) signal segments were extracted from rats subsequent to the injection of the inhibitor or the control solution, with a focus on epileptic seizure states (Fig. S7A and B). In Rat A, the Sv DC waveform exhibited high-amplitude variability during both the early (45 to 75 min) and late (75 to 105 min) phases following the control injection. However, after the inhibitor was injected into Rat A, the amplitude of fluctuations in each channel during seizures was significantly reduced (Fig. S7C). A comparison of the dispersion and mean root mean square values of the signals during the early and late phases of the control injection revealed no significant difference between the 2 phases (Fig. S7D). In contrast, intraperitoneal injection of HC-030031 significantly decreased the Sv DC field features in the late phase compared to the early phase (Fig. S7E and F), this phenomenon observed in both Rat A and Rat B. Analysis of the temporal distribution in Rat A showed that after injection (indicated by the dashed line), the trends in dispersion and power shifted from stable fluctuations to a decline over time (Fig. S7G and H). Rat B, which received only the inhibitor injection, exhibited a pattern consistent with that of Rat A: Following drug injection, Sv DC dispersion and fluctuation power gradually decreased over time (Fig. S7G to J), indicated by the orange regions. These data imply a temporal precedence of hippocampal astrocytic Ca^2+^ signaling prior to seizure onset, suggesting a correlative rather than a causal relationship.

## Discussion

Our prior work introduced the concept of the Sv DC field as a novel biophysical entity underlying large-scale cortical dynamics [13]. In the current study, using intracranial recordings and intraoperative cortical monitoring in epilepsy patients, we show that during visual, motor, and language tasks, the Sv DC field exhibits large-scale convergence and suppression, along with information transfer to other EEG frequency bands. We also observed consistent Sv DC waveforms in macaque, rat, and mice models of seizures, highlighting that this phenomenon is present across mammalian species. Further studies in mouse models revealed that prior to epileptic seizures, the dynamic changes in Sv DC potentials exhibit a temporal correlation with Ca^2+^signaling in astrocytes. Our findings reveal that the Sv DC field integrates large-scale cortical network activity with region-specific modulation, offering a potential novel target for interventions in epilepsy disorders.

### Cross-species conservation of Sv DC properties

Previous reports of ictal DC shifts, cortical slow potentials, or ultraslow activities described signal characteristics at the phenomenological level—typically as voltage drifts at single-electrode sites or temporal rhythmic features below 0.5 Hz [6,20,24,25]. These terms lacked an explicit physical basis. We have defined the Sv DC field strictly according to Maxwell’s electromagnetic theory: a macroscopic DC electric field existing in the 3-dimensional space of the human brain, generated by slowly varying potential gradients across different cortical depths, brain regions, and subcortical structures. This definition elevates prior 2-dimensional signal descriptions to a genuine biophysical entity. By unifying various slow electrical activities under the coherent concept of the 3-dimensional Sv DC field, we have resolved long-standing inconsistencies in terminology and elucidated their physical properties, thereby integrating decades of diverse observational findings in this field within a unified framework.

In addition to recordings from human subjects, mice, and rats, we also detected consistent Sv DC waveforms in a macaque model of acute kainic acid (KA)-induced seizures. The electrophysiological data obtained from macaques exhibited the same pattern observed in human and rodent subjects: prominent ultralow-frequency fluctuations emerged at seizure onset, and the enhancement of the Sv DC field occurred prior to the emergence of conventional high-frequency discharges. Despite the limitation that this observation was obtained from only 1 experimental animal and focused on seizure states without concurrent Ca^2+^ imaging, the findings indicate that the Sv DC phenomenon is not restricted to rodents or humans but is conserved across multiple mammalian species, which provides empirical support for the phylogenetic conservation of this electrophysiological feature.

### Large-scale changes in Sv DC fields and their relationship with task complexity

In the current study, using intracranial recordings and intraoperative cortical monitoring in epilepsy patients, we showed that during visual, motor, and language tasks, the Sv DC field exhibits large-scale convergence and suppression, along with information transfer to other EEG frequency bands. Regionally, visual areas display rhythmic Sv DC oscillations tightly coupled with gamma activity and increased power, whereas nonvisual areas show reduced Sv DC power. In language cortices, Sv DC oscillations follow spatiotemporal patterns consistent with known functional anatomy. Collectively, during brain information processing, Sv DC fields show unique large-scale change patterns that correspond closely to functional demands during different tasks.

From an electrophysiological perspective, the convergence of local and large-scale Sv DC components indicates reduced voltage differences leading to suppressed electromagnetic fields at both local and large scales. This phenomenon can be interpreted as the brain’s active adjustment of its “magnetic field” environment during tasks to meet varying information processing demands. The large-scale Sv DC field suppression likely reflects decreased whole-brain synchronization alongside enhanced specialized activity in the local functional cortex. This adjustment facilitates more focused and precise processing of task-relevant information. This mechanism resembles the reduction of unnecessary signal interference and transmission resistance within a unified magnetic field environment and enables efficient regional communication through complex adaptive mechanisms and robust signaling protocols.

Further research revealed that the degree of Sv DC field convergence varied with task complexity. For example, more pronounced Sv DC convergence and depression were observed during fine hand movement tasks. Similarly, compared with relatively simple-naming tasks, repetition tasks, which involve multiple cognitive processes, including language comprehension, memory, and expression, presented stronger Sv DC convergence. These findings might suggest that during complex tasks, the brain requires the Sv DC field to maintain a focused and sensitive state for more precise information processing.

### Local oscillation characteristics of Sv DC fields and their fine regulatory roles

Although large-scale alterations in Sv DC fields are notable, their local oscillations within specific brain regions provide distinctive perspectives on information processing. During visual tasks, Sv DC suggests rhythmic oscillations in visual areas, which unveil a finely-tuned regulatory mechanism. These oscillations possess 3 crucial characteristics: (a) Spatial specificity, which is only observed in vision-related regions, suggesting a strong coupling with regional function. (b) Phase-locking, where peak potentials are temporally aligned with the onset/offset of the stimulus, reflecting real-time brain responses. (c) Hierarchical dependence-lower-level areas (e.g., V2) exhibit stronger oscillations compared to higher-level areas, which is consistent with the known high-frequency responses (Fig. 5 and Fig. S5). This implies that low-frequency fields can effectively track local neural activity.

Notably, neighboring brain regions exhibit opposing oscillation directions, with inverted peaks positioned across cortical sulci (Fig. 5E). This indicates the capacity of Sv DC to capture the directionality of the local field, providing insights into interneuron communication and brain plasticity. By utilizing TE to analyze the directional information flow during visual tasks, the following observations were made: (a) Within 0 to 300 ms after the onset of the stimulus, the TE was most stable and reached its highest level; (b) in comparison to the blank-screen periods, the mean TE increased significantly. Therefore, Sv DC not only participates in local activation but also mediates interregional information transfer and coupling.

Analogous rhythmic oscillations were identified in motor and language tasks (Figs. 5 and 6). These oscillations were more prominent during intricate movements, nonexistent during simple repetitive movements, and diminished as task proficiency advanced. These discoveries underscore the role of Sv DC in local processing and large-scale network regulation. These outcomes also provide electrophysiological evidence for brain plasticity. When the original functional areas are impaired, the surrounding cortical regions may reorganize functional coordination within the large-scale functional networks [26]. Furthermore, time–frequency ITPC analysis across 6 patients performing visual tasks revealed that electrodes in visual functional regions exhibited markedly stronger task-locked phase coherence than nonvisual regions, supporting the specificity of Sv DC modulation to functionally engaged cortical areas.

### Distribution of power in the Sv DC field and large-scale functional regulation of the brain

During brain information processing, Sv DC fields are crucial for coordinating information transfer between brain regions. Spatially, spectral power increase is specific to visual-related brain areas (Fig. 5). Temporally, after a visual task starts, power in different frequency bands in functional areas changes dynamically. In the early task phase (100 to 150 ms), theta and alpha band activity increases most prominently, followed by stronger DC and delta band activity. The spectral power increase peaks around 300 to 350 ms and then declines. This temporal progression matches the spatiotemporal evolution of ERP signals. Intracranial and extracranial electrical recordings show synchronized physiological activity, and the composite signal of DC and other frequency bands follows ERP waveform patterns. This is consistent with Genkin et al.’s [27] finding in the primate PMd. After task onset, both low-and high-frequency power levels increase compared to the resting state, with low-frequency power having a larger proportion. The DC component reshapes local field potentials by injecting power into high-frequency bands (>30 Hz), with spatial specificity. TE analysis confirms directional information flow between DC inputs and ERP variations, indicating DC’s role in regulating local neural population energy metabolism through nonlinear coupling [27]. The characteristics of Sv DC fields add the new evidence for explaining the dissociation between attractor dynamics and geometric representations.

During task performance, distant task low-response associated region areas showed neither functional activation (no high-frequency activation changes) nor direct Sv DC inputs. However, they exhibited a significant reduction in Sv DC power (ΔDC/DC₀ = −0.59 ± 0.30). This passive response pattern suggests that the DC system may regulate functional networks through large-scale field potential modulation. The intense fluctuations in spectral power within this task low-response associated region provide evidence for the large-scale regulation of functional networks by the Sv DC field.

### Sv DC fields and the hierarchical management of brain function

The regulatory impacts of Sv DC fields on large-scale brain function contribute to the theoretical models of hierarchical brain management. Conventional theories suggest that the brain’s hierarchical functional architecture stems from specific neural connectivity patterns, and that structure–function coupling serves as the basis for efficient information processing. Lower-level regions (e.g., the primary visual cortex) demonstrate dense subcortical white matter connections, forming specialized sensory-motor processing units. Conversely, higher-level areas (e.g., the default mode network) present sparser long-range connections that facilitate cross-modal integration and abstract cognition [28]. Our research indicates that in lower-level visual cortical areas, the Sv DC exhibits distinct task-dependent rhythmic oscillations, while higher-level brain regions lack this feature. We hypothesize that the Sv DC field exerts large-scale regulatory effects on brain networks by transferring information across frequency bands and coordinating large-scale power distributions. In accordance with biophysical field properties, Sv DC-mediated information transfer and spectral power allocation are not restricted by anatomical white matter pathways. During complex tasks, Sv DC fields utilize phase coding and cross-frequency coupling to achieve spatiotemporal multiplexing of functional networks, enabling transmission across white matter tracts and substantially enhancing the information transfer efficiency. The discovery of Sv DC fields not only offers electrophysiological evidence for this hierarchical management but also overcomes the limitations of traditional structure–function coupling frameworks.

At the neurophysiological level, the Sv DC field offers an explanatory framework for the brain-splitting results reported by Schaefer et al. [29], supporting the notion that it comprises multiple relatively independent subfields that perform different functions. When complex information surpasses the capacity of the main functional areas, the Sv DC field, as a larger underlying basic field, participates in information processing and transmits information to subsequent associative and higher-level functional areas. In this process, rhythmic oscillations of the Sv DC field in the functional areas can be detected, while the characteristics of the Sv DC field outside these areas gradually converge to promote effective information transmission and sharing. This process establishes the necessary neurophysiological foundation for large-scale information integration and collaborative processing in the entire brain, enabling efficient comprehensive analysis, reasoning, and judgment of complex information. Once the brain is fully adapted to complex tasks and transforms them into proficient tasks, the involvement of the Sv DC field will be correspondingly reduced, allowing the brain to prepare for processing new information. This electric field integration mechanism reveals the fundamental characteristics of the brain’s functional network: Its ability for dynamic reorganization depends not only on fixed anatomical connections but also on real-time electric field modulation, thereby realizing a “software-defined” functional network. This regulatory mechanism shares similar technical principles with software-defined radio [30]. This dynamic functional modulation model provides a reference framework for the further development of brain–computer interfaces.

### Significance of the Sv DC in brain connectivity from a large-scale perspective

In the realm of brain connectivity research, investigations are generally classified into microscale, mesoscale, and macroscale approaches based on the research subjects and spatial resolution [31]. The microscale focuses on the connections between neuronal synapses, whereas the mesoscale mainly examines the connectivity among neuronal assemblies (e.g., cortical columns and neural nuclei) [31]. Owing to the current limitations of brain imaging technology, fully uncovering the complex connectivity patterns and functional mechanisms at these 2 scales remains a formidable challenge. The macroscale treats brain regions as fundamental functional units and explores the connectivity between different areas. This approach enhances the understanding of how various brain regions collaborate in cognitive, emotional, and behavioral processes and offers a comprehensive perspective on the brain’s functional organization, information transmission, and integration mechanisms. A notable contribution of this study is its demonstration that the Sv DC can serve as a novel signal marker to reveal the functional connectivity patterns between different brain regions.

Conventionally, fMRI technology based on BOLD signal changes has been predominantly employed to study the functional connections between brain regions [32,33]. Nevertheless, the Sv DC may reflect another crucial electrophysiological activity pattern in the brain, complementing the metabolic activity indicated by the BOLD signal. By investigating the distribution and dynamic changes of the Sv DC in different brain regions, functional connection patterns that are difficult to detect using traditional methods can be identified, thereby achieving a more comprehensive understanding of the functional network architecture of the brain.

The brain’s functional network is not static but can dynamically reorganize in accordance with different task requirements and environmental changes. By monitoring the real-time Sv DC changes in various brain regions, the dynamic adjustment of functional connections during different cognitive tasks can be observed. This dynamic network reorganization may involve the recruitment of new brain regions into the functional network or the modification of the connection strength between existing regions. Therefore, the Sv DC field provides a novel tool for studying brain network connections at the macroscopic level and a new method for understanding dynamic network reorganization. These findings offer novel perspectives for comprehending the pathogenesis of diseases and developing innovative regulatory therapies.

This study holds significance as it posits that the Sv DC field can serve as a bridging instrument for cross-scale brain connectivity research (Fig. 8). By integrating macroscale Sv DC-related functional connectivity with mesoscale neuronal ensemble connections, we can explore the neural circuit basis underpinning the functional connections between brain regions. Furthermore, when combined with microscale synaptic connection mechanisms, this approach enables a deeper understanding of the structure–function relationship in the brain. Thus, the Sv DC field provides both a new tool for investigating brain network connectivity at the macroscopic level and a new approach for understanding dynamic network reorganization. These findings provide new perspectives for understanding disease pathogenesis and developing innovative regulatory therapies.

**Fig. 8.**
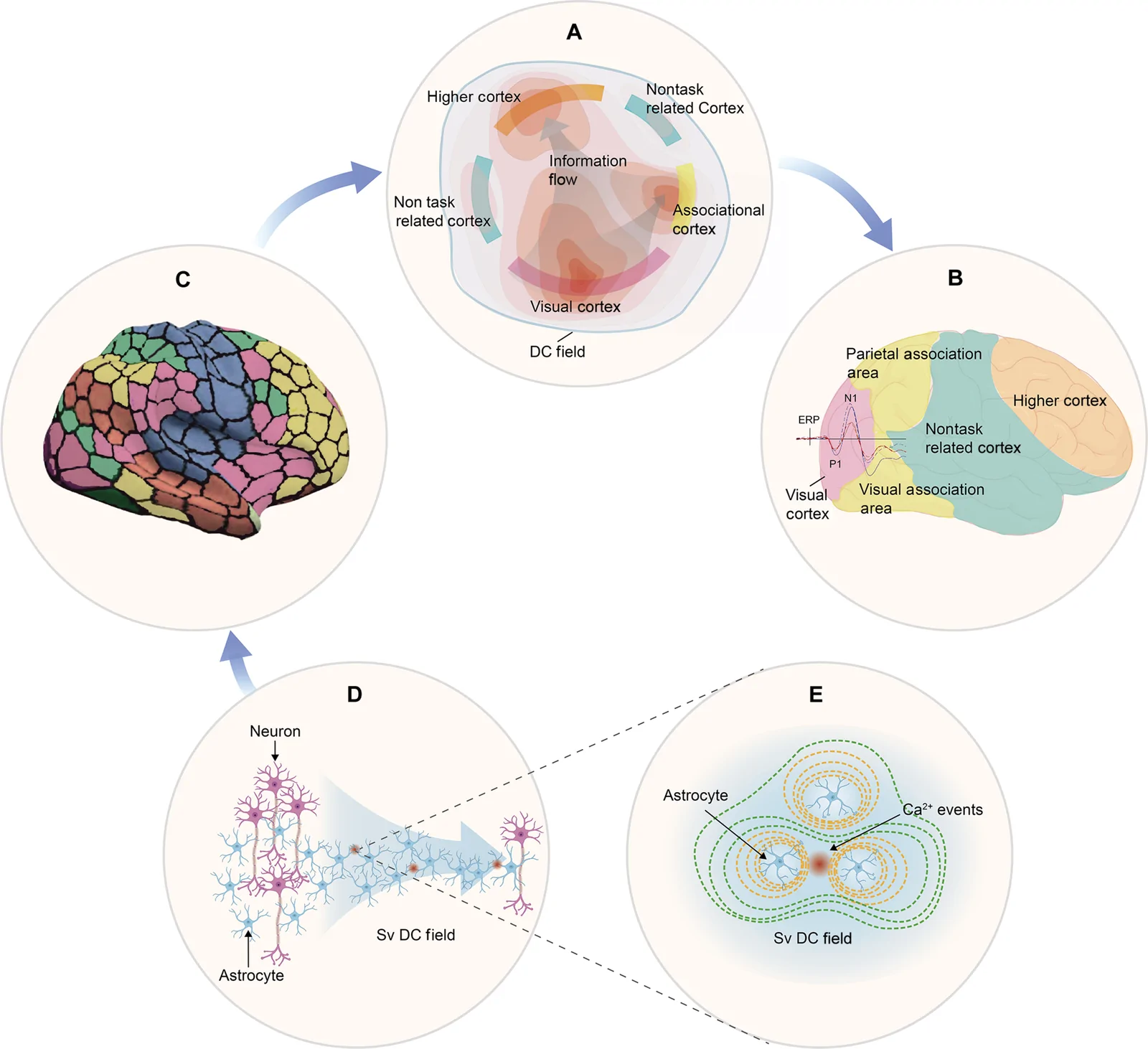
Schematic diagram of the hypothesized slow-varying direct current (Sv DC) field (from micro-generation to macro-modulation). (A) Illustration of the Sv DC field patterns among functional areas, the association cortex, the higher-level cortex, and task low-response associated region regions. Each region sustains its own independent working field, as depicted by the concentric, honeycomb-like red patterns encircling different cortical territories in the figure. When functional areas are engaged in task execution, the Sv DC field promotes whole-brain power redistribution to support task-relevant regions. Moreover, when the task necessitates multiregional collaboration, the functional areas expeditiously transmit information to higher-level brain regions via the Sv DC field. (B) Illustration of the hierarchical functional coordination among functional areas, the association cortex, and the higher-level cortex at the macroscopic level. Taking event-related potentials (ERPs) as an instance, the early physiological waves within the initial 100 ms are predominantly generated by the visual cortex in the occipital lobe. This information is then transmitted through the Sv DC field to higher-level brain regions for subsequent visual processing. (C) Illustration of the modular “honeycomb-like” organization of the human cerebral cortex. This panel is adapted from Fig. 4 in [29], which presents a parcellation of the cortex into distinct functional modules based on resting-state functional magnetic resonance imaging (fMRI) connectivity. The homogeneous coloring of parcels reflects their shared network identity, highlighting how functionally coherent areas are spatially segregated yet collectively form a densely packed, tiled arrangement resembling a honeycomb structure. This organizational pattern supports the notion that distinct cortical regions occupy dedicated and spatially segregated territories. (D) An illustration of the mesoscopic-scale mechanism through which functional areas rapidly relay information to other higher-level cortices via the Sv DC field maintained by neuron–astrocyte networks. (E) An illustration of the microscopic-scale maintenance of the Sv DC field through calcium events among astrocytes.

### Sv DC fields and the energy consumption of the brain

Understanding the role of the Sv DC field within large-scale functional brain networks might provide further insights into brain energy consumption. Taking language function as an example, during an auditory comprehension task with a covert response, DC oscillatory activity can be detected in Wernicke’s and Broca’s areas. This corresponds spatially to the regions of increased BOLD signals observed via fMRI, suggesting that the functional activation of localized brain regions is correlated with energy consumption (Fig. 6B).

However, this phenomenon does not account for the primary component of the brain’s overall energy expenditure. Utilizing connectome-based neuroimaging to calculate the energetic cost of signal transmission within functional networks, Castrillon and colleagues found that the frontoparietal network demands approximately 67% more energy per gram than the sensorimotor network [34]. This finding implies that human cognitive evolution may be driven not only by an increase in brain volume but also by the development of slow-acting neuromodulatory circuits.

Furthermore, the current research suggests that, compared to the high amplitude and degrees of freedom characteristic of Sv DC activity in the resting state, task execution induces a marked convergence of Sv DC fields across different brain regions. Whether this modulation of large-scale functional networks, manifested by Sv DC fields, is associated with the brain’s substantial energy consumption remains a question of profound scientific interest. Naturally, this hypothesis awaits further validation through more supportive evidence from studies coupling cerebral metabolic imaging with electrophysiology.

### The potential contribution of astrocytes in Sv DC fields

The cellular sources of the low-frequency potentials described in this study remain unresolved. Astrocytes represent one of several possible contributors to infraslow electrophysiological activity, but the present data do not identify a specific cellular generator of the Sv DC component. Over the past few decades, astrocytes were regarded as electrically nonexcitable, establishing isopotential syncytia through direct gap junction coupling [35]. Nevertheless, mounting evidence currently indicates that astrocytes exhibit “electrical activity”. They sustain hyperpolarized membranes and express voltage-gated channels [36], and their potential syncytia are capable of establishing large-scale communication networks [37,38]. Studies have demonstrated that when astrocytes encode information via Ca^2+^ event patterns, their spatial activity patterns can change instantaneously. Since Ca^2+^ transients are triggered sequentially across different cells, Ca^2+^ signals propagate in a wave-like manner through astrocytic networks [39]. Research utilizing the AstroMEA technology to differentiate astrocytic and neuronal network functions has shown that astrocytic networks do not transmit signals in a purely linear manner [40]. These findings provide a basis for future investigation, but they do not demonstrate that astrocytic activity generates or maintains Sv DC signals.

Astrocytic Ca^2+^ activity may occur in temporal association with changes in conventional electrophysiological signals during pathological states. However, such temporal associations do not establish that astrocytes are the source of Sv DC activity or that astrocytic signaling directly regulates its dynamics. In addition, measurements of astrocytic Ca^2+^ activity and Sv DC signals may involve different experimental preparations, recording modalities, and signal-processing procedures. Their relationship should therefore be interpreted cautiously.

Pharmacological studies involving systemic modulation of channels expressed by astrocytes also cannot provide cell-type-specific evidence. Changes in low-frequency signal features may reflect effects on multiple cell populations or other physiological processes, including neuronal activity, seizure evolution, vascular responses, anesthesia, or systemic drug actions. Accordingly, these observations should not be interpreted as evidence for an astrocyte-specific mechanism.

Astrocytic network studies may offer a conceptual framework for future research, but similarities between astrocytic network organization and the spatial distribution of Sv DC signals do not establish a causal relationship. Direct evaluation would require simultaneous recording of Sv DC signals and validated cell-type-specific activity, together with selective manipulation of astrocytes and appropriate controls for neuronal, vascular, seizure-related, and movement-related influences.

The possible contribution of astrocytes to Sv DC signals therefore remains an open hypothesis. The present evidence is insufficient to support the view that astrocytes generate, maintain, or act as a principal regulator of the Sv DC field, and no direct inference should be made regarding astrocyte-mediated mechanisms of human cognition or consciousness.

### Limitations

This study encompasses several limitations that merit recognition. Firstly, the sample size was comparatively small (*n* = 12), which might restrict the generalizability of the findings. Simultaneously, the limited number of cases under each task condition constrained the capacity to statistically affirm this consistency. In addition, no synchronously hardware-acquired electrooculography/electromyography recordings were obtained during SEEG data collection; eye movement and motion artifacts were excluded solely based on post hoc waveform characteristics. Moreover, this study predominantly focused on macroscopic electrophysiological phenomena and put forward a hypothetical mechanism underlying Sv DC modulation; however, further validation at the cellular or circuit level is still requisite. Specifically, the rodent seizure experiments reported herein are exploratory and correlational and do not establish a causal role for astrocytes in generating Sv DC fields. The macaque data are derived from a single animal and are limited to seizure states without concurrent Ca^2+^ imaging and thus should be interpreted as preliminary observational evidence rather than confirmatory findings. Together, these animal data support an association between astrocytic activity and Sv DC dynamics but do not directly substantiate a causal mechanism in human cognitive tasks. Future studies integrating direct manipulation of astrocytic activity with simultaneous Sv DC recordings, across multiple species and including both physiological and pathological states, are necessary to test this hypothesis and elucidate the underlying mechanisms.

## Conclusion

In this study, we characterized the Sv DC field as a macroscopic electrophysiological phenomenon that exhibits both large-scale and region-specific modulation across different functional states. During visual, motor, and language tasks in epilepsy patients, the Sv DC field showed large-scale convergence and suppression, with local task-dependent rhythmic oscillations in functional areas and power redistribution—increasing in task-active regions while decreasing in task low-response associated region areas. During seizures, Sv DC dynamics displayed characteristic spreading patterns that were consistently observed across humans, macaques, rats, and mice, suggesting a phylogenetically conserved phenomenon. In rodent models, these dynamics were temporally correlated with astrocytic Ca^2+^ signaling, though the relationship remains correlational and requires further mechanistic validation. Taken together, these findings suggest that the Sv DC field may serve as a large-scale regulatory mechanism for hierarchical functional organization in the mammalian brain, complementing traditional structure–function coupling frameworks.

## Materials and Methods

### Patient selection and intracranial recording

Patients with drug-resistant epilepsy who underwent presurgical evaluation and subsequent surgical treatment at the Beijing Institute of Functional Neurosurgery between November 2024 and April 2026 were enrolled. The inclusion criteria were as follows: (a) implantation of SEEG electrodes in visual, language, or motor functional areas; (b) functional testing during the preoperative evaluation or intraoperative mapping to ensure surgical safety; (c) the patients demonstrated good capacity to cooperate with and complete the corresponding functional tasks, with less than 10% of the total trials being excluded for various reasons; (d) no frequent interictal epileptiform discharges in the electrode contacts located in functional areas, not the seizure onset zone, and no significant recording artifacts; and (e) more than 2 h before and after any seizure during task testing. The participant inclusion and exclusion flowchart is shown in Fig. S8. This study was conducted in accordance with the principles of the Declaration of Helsinki and was approved by the ethics committee of Xuanwu Hospital, Capital Medical University (approval no. LYS[2024]297-001).

A total of 12 patients were included in this study, comprising 7 males and 5 females, with a mean age of 21.75 ± 7.09 years. Among these 12 patients, 6 with SEEG electrodes implanted in the occipital lobe underwent visual paradigm testing. Three patients with SEEG electrodes implanted in the precentral gyrus participated in a hand movement paradigm test. Three patients with SEEG electrodes implanted in the language functional area completed language function testing, including 1 patient (Patient J, female, 17 years old) who underwent intraoperative awake language mapping. Detailed clinical information is presented in Table 1.

**Table 1.** Demographic and clinical data for the patient cohort

Pt	Gender	Age (years)	Epilepsy duration (years)	MRI lesion location	Seizure type	Medication (ASMs)	Electrodes/Contacts	Lead in SOZ	Surgery	Task
Y	M	36	14	N	Focal	LTG, OXC	8/108	L OL	RF: L OL, Hip, Amy	Visual
F	M	21	12	N	sGTCS	VPA, LTG, TPM, PER	13/198	R PL, OL	RF: R PL, OL, TL	Visual
W	F	24	8	L CR	Focal	OXC, LEV	6/92	L CR	RF: L CR	Motor
A	M	30	15	N	sGTCS	VPA, PB, OXC	11/164	L TL	RF: L Hip, Amy, TL	Language
J	F	17	9	N	sGTCS	LEV, PER, OXC	11/142	L IL	RS: L IL	Language (intraoperative awake)
L	F	10	4	N	sGTCS	LTG, VPA	12/188	R PL, OL	RS: R PL	Visual
P	F	21	9	L Hip	Focal	LEV	7/102	L Hip, Amy	RF: L TL, IL	Language
S	M	16	6	N	sGTCS	OXC, PER	10/106	R CR	RF: R CR	Motor
T	F	15	6	N	sGTCS	OXC, LCM	10/144	L FL	RF: L FL	Motor
R	M	27	15	N	Focal	OXC	11/156	L PL	RF: L PL	Visual
H	M	20	10	N	sGTCS	PB, PER, LEV, LCM	10/158	R OL	RF & RS: R OL	Visual
C	M	24	22	L TL	sGTCS	OXC, PER	11/166	L TL	RF: L TL	Visual

Pt, patient; ASMs, antiseizure medications; M, male; F, female; N, negative; R, right; L, left; sGTCS, secondary generalized tonic-clonic seizure; RF, radiofrequency ablation; RS, resection; TL, tempolar lobe; PL, parietal lobe; OL, occipital lobe; IL, insular lobe; CR, central region; Hip, hippocampus; Amy, amygdala; SOZ, seizure onset zone; LTG, lamotrigine; OXC, oxcarbazepine; VPA, valproate; PB, phenobarbital; TPM, topiramate; PER, perampanel; LEV, levetiracetam; LCM, lacosamide

Immediately following the SEEG electrode implantation, patients underwent long-term intracranial video-EEG monitoring using the Neuracle NSH0256 digital EEG system (sampling rate: 2,000 Hz). The monitoring duration ranged from 3 to 14 d, with the objective of recording at least 2 habitual clinical seizures per patient. For patients with infrequent seizures, medication could be appropriately reduced or discontinued under the guidance of a physician. For each patient undergoing video-EEG monitoring, a comprehensive analysis of all EEG data during both the interictal and ictal periods, along with seizure symptoms and noninvasive examination results, was carried out by at least 3 electrophysiological specialists and neurosurgeons. This analysis aimed to precisely locate the seizure onset zone and the propagation areas of each patient’s seizures.

### Task paradigms

#### Visual function testing

The visual task was executed using a 16-inch laptop monitor with a viewing distance of approximately 60 cm and a black background. Each trial commenced with a 3-s display of a black screen, succeeded by a 1-s presentation of a stimulus screen featuring a gray background. During the display of the stimulus screen, a white boat-shaped image either randomly emerged at 1 of 8 potential locations on the screen (i.e., top, bottom, left, right, upper-left, lower-left, upper-right, or lower-right) or did not appear at all. The target locations and the presence or absence of the stimulus were determined in a fully randomized fashion, with each of the 8 positions presented 10 times to guarantee a balanced spatial distribution. Following each stimulus presentation, a 3-s black screen served as an intertrial interval and recovery period. This cycle was repeated throughout the task, and participants were instructed to fixate on the center of the screen throughout. When the white boat image appeared, they were required to promptly shift their gaze to the target and visually track its location. Patients participating in the experiment underwent 10 trial training sessions prior to the formal data collection to ensure they fully understood the task before the formal experiment began. SEEG data were collected from each participating patient over a total of 89 trials.

#### Motor function testing

The motor task was carried out under the guidance of a clinician. Participants were instructed to perform 3 types of movements in accordance with the clinician’s verbal commands: (a) a simple repetitive movement commonly observed in daily life, fanning with the right hand at a frequency of approximately 2 times per second for 1 min; (b) a fine repetitive movement involving transferring all beans from one bowl to another using a spoon with the right hand, until the entire bowl of beans had been moved; and (c) a gesture-based task involving the sequential display of hand gestures to represent the numbers 1 through 10 using the right hand, performed once per trial. All participants were right-handed.

#### Language function testing

fMRI-Based Language Task: The auditory comprehension task employed during fMRI incorporated an auditory version of a word-definition paradigm. In each trial, patients passively listened to approximately 10 to 16 Mandarin words describing common daily-life objects. During the brief pauses between words, participants were instructed to silently judge whether the previously mentioned item was animate or inanimate. The number of words and the duration of the interword pauses varied slightly according to the participant’s educational background. During 3 20-s control trials, participants only heard background scanner noise.

EEG-Based Language Task: The EEG-based language tasks included picture naming and question answering. The picture-naming task was conducted using a 16-inch laptop monitor with a viewing distance of 60 cm. A total of 9 full-color images of common everyday objects (e.g., refrigerators, pencils, and cars) were selected to ensure that all participants had basic familiarity. All images were standardized in terms of color, resolution, and size to minimize the influence of low-level visual features. The images were presented passively in sequence, with each image displayed for 3 s and automatically transitioning to the next without participant intervention. Participants were instructed to name the object aloud immediately after each image appeared. The task was performed in a quiet environment to ensure optimal attention and minimal external distraction. The question-answering task consisted of 8 short, simple daily-life questions, each fewer than 10 Chinese characters in length. The questions covered common-sense or time-related information with a single correct answer (e.g., “What day is it today?” “How many months are there in a year?”). All questions were presented via audio playback in standard Mandarin at a normal speaking pace. The recordings were played in a quiet environment to ensure clarity and moderate volume. Participants were asked to respond orally as soon as they heard each question. A physician was present to supervise the session and assess the accuracy of the participants’ responses.

Intraoperative Awake Language Task: Participants who required intraoperative language mapping under awake conditions were positioned in a lateral decubitus posture. After awakening, they performed the same picture-naming task described above as well as a sentence-repetition task. The sentence-repetition task consisted of 10 simple, easily understood Chinese sentences, each exactly 7 characters in length (e.g., “The weather is nice today”). All sentences were played on a computer in standard Mandarin at a normal speaking rate to ensure consistent and intelligible auditory input. After each sentence, participants were asked to repeat it as accurately as possible.

### Data processing and feature calculation

#### EEG signal processing

EEG Signal Preprocessing and Data Selection: The raw EEG data underwent preprocessing via digital filters. Initially, the notch operation was executed using the filter in the EEGLAB toolbox, with notch frequencies set at 50, 100, 150, 200, and 250 Hz, respectively. Subsequently, a 0.01- to 249-Hz band-pass Butterworth filter (fourth-order filter) was employed to preserve signals within the effective frequency range. All channels were then visually inspected for artifact identification and screening. Channels demonstrating significant baseline drift (continuous amplitude shifts exceeding ± 100 μV) or noise resulting from poor electrode contact (e.g., sudden high-amplitude fluctuations or signal discontinuities) were marked as invalid and excluded. The remaining channels without observable artifacts (e.g., electromyographic interference, eye movements, or motion artifacts) and with stable signals were retained as valid data for analysis. All preprocessing procedures were implemented using the EEGLAB toolbox (version 2021.1), and cross-validation was independently performed by 2 researchers to ensure consistency.

Sv DC Component Filter Design: To isolate the 0.01- to 0.5-Hz DC component from brain electrical signals, a fourth-order filter Butterworth band-pass filter was utilized, and zero-phase filtering was implemented using the filtfilt() function in MATLAB. The filtfilt() function implements zero-phase correction based on the algorithm proposed by Gustafsson [41]. To validate the performance of the fourth-order Butterworth filter in controlling edge effects during ultralow frequency band-pass filtering, and to assess its behavior when transient events occur, we conducted synthetic signal recovery tests and compared filters of various orders. Detailed testing methods can be found in the supplementary materials. The fourth-order Butterworth filter and zero-phase filtering method used in this study satisfy the expected filtering requirements, with the oscillation time of signal attenuation at the beginning and end of the signal lasting less than 1 min. Whether during rest or task periods, the waveforms obtained after filtering at 0.01 to 0.5 Hz reflect the true low-frequency slow-wave trends rather than being influenced by transient potentials. In the actual filtering process, the complete SEEG dataset is used for filtering and analysis. The complete SEEG dataset includes more than 10 min of resting-state data collected before and after the task period. After filtering is complete, the excess portions at the beginning and end, which were not used for analysis, are removed to further reduce edge effects.

Although the filter exhibits a gradual roll-off rather than an abrupt cutoff, signals beyond this frequency are attenuated, retaining only minimal residual power, which does not affect the overall trend of the results. After the original brain electrical signals were input into this filter, the Sv DC component within the desired frequency band was retained, while high-frequency noise above 0.5 Hz was filtered out. This design facilitated the extraction of the low-frequency Sv DC component in the brain electrical signals for subsequent analysis.

#### Average power for SEEG signals

To quantitatively assess the intensity of SEEG recordings, we utilize the mean square value of the signal as a direct and robust metric for its average power. Under the assumption of a normalized unit impedance (1 Ω), the instantaneous power of the signal is proportional to the square of its instantaneous amplitude. Consequently, the average power over a specific time epoch is obtained by averaging these squared instantaneous values over the observation interval. In practice, for discrete sampled SEEG data *x*[*n*] of finite length *N*, the average power is computed as the sample mean of the squares:

$$P_{\mathrm{avg}}=\frac{1}{N}\sum_{n=0}^{N-1}\;{\left|x\left[n\right]\right|}^{2}$$ (1)

#### ITPC calculation

The ITPC was employed to assess the phase consistency across trials for each EEG channel, specifically reflecting the degree to which the instantaneous phase of the signal at a given time point is locked to the experimental event [42,43]. For an EEG dataset consisting of *M* trials, the ITPC for channel *c* at time point *t* (within a specific frequency band) was calculated using the following formula:

$${\text{ITPC}}_{c}\left(t\right)=\frac{1}{M}\left|\sum_{m=1}^{M}e^{i\theta_{c,m}\left(t\right)}\right|$$ (2)

Here, $\theta_{c,m}\left(t\right)$ denotes the instantaneous phase at time *t* for channel *c* in the *m*-th trial, obtained via the Hilbert transform (or complex Morlet wavelet transform) on the filtered signal. The ITPC ranges from 0 to 1, where higher values signify stronger phase locking across trials and more consistent phase alignment relative to the onset of the stimulus. In this study, we computed the ITPC for each channel across all trials to quantify the temporal reliability of the neural responses.

#### Transfer entropy calculation

TE is an information measurement method derived from mutual information theory [44]. Consider that random processes *X* and *Y* generate 2 simultaneously measured time series x_t_ and y_t_. The delay embedded vector is defined as $x_{t}^{d}=\left(x_{t},{\;x}_{t-\tau },{\;x}_{t-2\tau },\cdots ,{\;x}_{t-\left(d-1\right)\tau }\right)$, where *d* is the dimension of the embedding space and *τ* is the delay. The same representation is used for the time series $y_{t}^{m}=\left(y_{t},y_{t-\tau },\cdots ,y_{t-\left(m-1\right)\tau }\right)$. The transfer entropy $\mathrm{TE}\left(Y\to X\right)$ from process *Y* to process *X* can be expressed as

$$\begin{array}{c} \mathrm{TE}\left(Y\to X\right)=H\left(x_{t+u}|x_{t}^{d}\right)-H\left(x_{t+u}|x_{t}^{d},\;y_{t}^{m}\right) \end{array}$$ (3)

where $H\left(*\right)$ is the entropy rate and *u* is the prediction time. The above formula can be further expressed by using the formula for conditional entropy:

$$\begin{array}{c} \begin{array}{c} \begin{array}{c} \mathrm{TE}\left(Y\to X\right)=H\left(x_{t}^{d},\;y_{t}^{m}\right)-H\left(x_{t+u},\;x_{t}^{d},\;y_{t}^{m}\right)+H\left(x_{t+u},\;x_{t}^{d}\right)-H\left(x_{t}^{d}\right) \end{array} \end{array} \end{array}$$ (4)

By representing the entropy value with probability, the following formula for calculating the TE can be obtained [44,45]:

TEY→X=∑pxt+uxtdytmlogpxt+uxtd,ytmpxt+uxtd (5)

In this study, the K-nearest neighbor approach was employed to estimate the TE [46]. Based on the suggestion from Kraskov et al. [46], the nearest neighbor retrieval quantity *K* was set to 4. The dimensions (*d* and *m*) and embedding delay (*τ*) were computed according to the Cao criterion [47], and the Theiler correction window (*T*) was set to 1 autocorrelation delay time as recommendation of Huang et al. [48]. The prediction time *u* was set to the minimum temporal resolution time, which was 0.5 ms in this investigation. In the actual calculations, this study used the Java Information Dynamics Toolkit (JIDT) to compute TE [49].

#### Language fMRI data processing

The fMRI data were preprocessed using SPM12 (Statistical Parametric Mapping, Wellcome Trust Centre for Neuroimaging, London) implemented on the MATLAB platform (MathWorks, Inc.). The preprocessing protocol included slice timing correction and head motion correction. Subsequently, T1-weighted anatomical images were segmented into gray matter, white matter, and cerebrospinal fluid and normalized to a standard template space. The derived normalization parameters were then applied to the functional images to spatially normalize them to the same standard space. Finally, the normalized functional images were smoothed using an 8-mm full-width at half-maximum Gaussian kernel. At the individual-subject level, statistical parametric maps of brain activation during the auditory comprehension task relative to the resting baseline were computed using an uncorrected voxel-wise threshold of *P* < 0.001.

### Neural signal acquisition in macaque

One adult male crab-eating macaque (*Macaca fascicularis*, aged 5 years, weight 4.6 kg) was enrolled in this study. Preoperatively, the subject underwent MRI and computed tomography scanning. The obtained MRI and computed tomography images were coregistered and subjected to 3-dimensional reconstruction, to determine the target site, implantation trajectory, and coordinate parameters for electrode placement in the primary motor cortex (M1) based on the individual brain anatomical structure and standard anatomical atlases. Electrode implantation was conducted under general anesthesia induced by 5% isoflurane. Guided by a stereotactic robotic system, a nickel-chromium alloy electrode was implanted into the M1 region in strict accordance with the preoperative imaging-based implantation plan. Postoperative routine care was implemented, and the subject was allowed a minimum 2-week recovery period to reduce the confounding effects of surgical trauma and acute postoperative reactions on subsequent intracranial EEG recording and epilepsy model establishment. After the recovery period, an acute epilepsy model was established via injection of 5 μl of KA into the M1 region. Following KA administration, intracranial EEG signals were continuously acquired using the Plexon OmniPlex system at a sampling rate of 1,000 Hz, and behavioral phenotypes of the subject were synchronously observed. Ictal EEG data were defined as epileptic electrical activity recorded within 2 h after KA injection. Only ictal EEG segments with qualified signal quality, free of obvious motion artifacts and electrode noise, were retained for subsequent analyses.

### Neural signal acquisition in rats

To elucidate the characteristics of the existing Sv DC field, a series of animal studies were designed and conducted on rats. Rats anesthetized with 1.5% isoflurane were fixed in a stereotaxic frame. After scalp incision, 18 cranial windows were drilled at ECoG probe coordinates, followed by a 3×3-mm bone window over the left hippocampal cortex. The ECoG probe was secured via micro-screws through cranial windows. A volume of 300-nl 4-aminopyridine was nanoinjected into hippocampal CA1 to induce seizures. A 16-channel microelectrode was implanted in the hippocampus, with reference/ground wires fixed near the cerebellum via additional cranial screws. To assess the contribution of astroglial calcium-dependent TRPA1 receptors to Sv DC field generation, HC-030031 or vehicle control was administered intraperitoneally 30 min prior to recording [50]. The details of Materials and Methods are presented in the Supplementary Materials.

### Neural signal acquisition for mouse Sv DC analysis

To characterize seizure-associated Sv DC activity in mice, hippocampal field-potential signals were recorded during chemically induced seizures. A 16-channel microelectrode array was implanted in the hippocampal CA1 region, and seizure activity was induced by local administration of 4-aminopyridine. Signals were acquired using a Neurostudio recording system (Changxiang Medical Limited) at a sampling rate of 30 kHz. The original acquisition files retained the 0.01- to 0.5-Hz components used for the Sv DC analysis shown in Fig. 7A and B. The detailed surgical and signal-acquisition procedures are provided in the Supplementary Materials.

### Statistical analysis

Descriptive statistics were initially computed for all variables, including the mean ± SD (for normally distributed data) or the median and interquartile range (for non-normally distributed data). The normality of the data was evaluated using the Shapiro–Wilk test in SPSS version 21.

To compare Sv DC power during tasks and at rest, we divided the first 1 s after task onset into 10 consecutive windows with a 100-ms step size, calculated the logarithmized mean square value for each window, and used the pretask 500-s isochronous resting period as the paired baseline. Next, construct the window energy difference $D_{c,i,j}$ for each trial:

$$D_{c,i,j}=\ln\left(E_{c,i,j}^{\text{Task}}\right)-\ln\left(E_{c,i,j}^{\text{Rest}}\right)$$ (6)

Here, *c* represents the electrode contact, *i* represents the window index (1 to 10), and *j* represents the trial number. A linear/quadratic regression model is established with the paired difference $D_{c,i,j}$ as the dependent variable and the trial number *j* (and the quadratic term) as the independent variables (if *P* > 0.05 for the quadratic term, it is reduced to a linear model). Use the intercept $\beta_{0,c,i}$ of the regression model as the trend-adjusted mean effect for that window, and extract its standard error to perform a 2-tailed 1-sided *t* test. Combine the *P* values from the 2-tailed, 1-sided *t* tests for all representative contact points and all windows (2 × 8 × 10 in total) into a single vector and apply Benjamini–Hochberg FDR correction uniformly.

In the comparison of TE across multiple time windows, a 1-way permutation repeated-measures ANOVA was used to test for main effects across 8 representative electrode contacts. For each electrode contact, 10,000 label permutations were performed (independently and randomly shuffling the order of groups within each experiment), and the *P* value of the observed *F*-statistic was calculated based on the permutation distribution. For multiple comparisons across the 8 contacts, the Benjamini–Hochberg FDR method was applied to correct for significant main effects, followed by paired permutation tests (10,000 permutations) to determine which specific group pairs exhibited differences. The raw *P* values for all post hoc comparisons (number of significant channels × 15 pairs) were again uniformly corrected using the FDR. Additionally, the paired Cohen’s *d* effect size was calculated, and a 95% confidence interval for the mean difference was constructed using the bootstrap percentile method (5,000 resamples).

## Ethical Approval

This study was conducted according to the principles of the Declaration of Helsinki and was approved by the ethics committee of Xuanwu Hospital, Capital Medical University (approval no. LYS[2024]297-001).

## Data Availability

The data that support the findings of this study are available from the corresponding author upon reasonable request.
