# Supplementary material for: The Sv DC Field: Large-Scale and Hierarchical Functions Regulator
Source: Research (Wash D C). 2026 Nov 13;9:1476. doi: 10.34133/research.1476 (PMC13760056; doi:10.34133/research.1476)
Supplement: Supplementary file 1 [file research.1476.f1.zip › Supplementary materials 1016.docx]

**Supplementary Figures and Legends**

**Fig. S1. Sv DC field dynamics during seizures in eight patients.** One representative seizure is shown for 8 patients in our study. The red dashed line marks the seizure onset.

**Fig. S2. The additional four patients also exhibited a converging and downward shift pattern in Sv DC during task performance.** For each of the four patients, the electrode implantation trajectory, the functionally relevant cortical region (Broca’s area in orange; precentral gyrus in blue; occipital cortex in purple), and the corresponding Sv DC traces during the task are shown. The red dashed lines indicate the task onset and offset. The upper two panels present Sv DC changes in two patients performing language tasks, while the lower two panels show Sv DC patterns in one patient performing a hand movement task and another performing a visual task.

**Fig. S3. The dynamic changes in the power difference between the task state and the resting state of Patient Y within the interval of 0 to 1000 milliseconds after autoregressive detrending processing.**

**Fig. S4. Correlation between transfer entropy and changes in power within the 0–300 ms window following a representative contact task.**

**Fig. S5. In another patient undergoing a visual task, task-related Sv DC oscillations were consistently observed at electrode contacts located in the visual cortical region.** This figure presents the visual task results from an additional patient. The upper panel shows the electrode implantation trajectory, the occipital functional region (indicated in purple), and the visual task paradigm. The lower panel displays the positions of selected representative contacts on MRI, along with their corresponding Sv DC traces recorded during task performance. Red dashed lines mark the onset and offset of each visual task block. Contacts located in the visual functional region are shown in green. The magnified view on the right highlights the detailed segment indicated by the grey-shaded area on the left.

**Fig. S6. Comparison of TE during the 0–0.3 time interval after the task begins with TE during daily state.**

**Fig. S7.**  **Changes in Sv DC of ECoG Signals in Rats During Seizures Following Injection of a TRPA1 Inhibitor.** **(a)** Multimodal epileptic signal recording. Field potential electrodes were implanted into the CA1 area of the hippocampus, and ECoG electrodes covered the entire cerebral cortex of rats. **(b)** Representative epileptic seizure signal and the waveform used for subsequent analysis. **(c)** The results of superimposing Sv DC waveforms on each channel, showing a randomly selected seizure segment in each group. (**d-f)** Statistical comparison results of two features—signal dispersion and mean channel RMS. (d: comparison of results in rat A during the early (45–75 min) and late (75–105 min) phases following the control injection, n = 12 Sv DC signals for 45-75 min group; n = 25 Sv DC signals for 75-105 min. e: comparison of results in rat A following injection of the inhibitory drug during the early (30–70 min) and late (70–100 min) phases, n = 27 Sv DC signals for 30-70 min group; n = 25 Sv DC signals for 70-100 min. f: comparison of results in rat B following injection of the inhibitory drug during the early (45–75 min) and late (75–105 min) phases, n = 10 Sv DC signals for 45-75 min group; n = 21 Sv DC signals for 75-105 min;.the rank-sum test, ns: no significant, **P* < 0.05, ***P* < 0.01, ****P* < 0.001). **(g-j)** Scatter plot showing how features change over time. The horizontal axis represents the sequence numbers of the epileptic seizure segments extracted in chronological order, and the vertical axis represents the feature values. g and h represent the distribution of results for rat A, while i and j represent the analysis of results for rat B. The blue background indicates the distribution of features following the control injection, and the orange background indicates the distribution of features following the injection of the inhibitory drug.

**Fig. S8. Participant inclusion and exclusion criteria flowchart for human studies .**

**Supplementary Tables**

**Table. S1. One-way repeated-measures permutation ANOVA (10,000 label permutations, shuffling time-window labels within each trial) on TE values.**

| Channel | F_statistic | p_FDR | Significance |
| --- | --- | --- | --- |
| A9 | F(5, 440) = 4.140 | 0.0021 | ** |
| B10 | F(5, 440) = 2.830 | 0.0176 | * |
| C10 | F(5, 440) = 1.591 | 0.1635 | n.s. |
| D9 | F(5, 440) = 8.117 | 0.0000 | *** |
| E10 | F(5, 440) = 6.425 | 0.0000 | *** |
| F11 | F(5, 440) = 4.209 | 0.0014 | ** |
| G10 | F(5, 440) = 2.361 | 0.0473 | * |
| H9 | F(5, 440) = 13.105 | 0.0000 | *** |

The degrees of freedom are (5, 440) for all analyses; significance is based on FDR correction (Benjamini-Hochberg method), α = 0.05; ** p < 0.01, * p < 0.05, n.s. indicates not significant.

**Table. S2. Results of Pairwise Post-Hoc Comparisons for Each Time Window on H9.**

| H9 Comparison | MeanDiff | CI_lower | CI_upper | Cohen_d | p_FDR | Significance |
| --- | --- | --- | --- | --- | --- | --- |
| -0.9~-0.6 vs -0.6~-0.3 | 2.1766e-03 | -4.5990e-03 | 8.7518e-03 | 0.0676 | 0.6536 | n.s. |
| -0.9~-0.6 vs -0.3~0 | 8.3790e-03 | 1.9513e-03 | 1.4686e-02 | 0.2714 | 0.0428 | * |
| -0.9~-0.6 vs 0~0.3 | -7.5880e-03 | -1.3687e-02 | -1.4781e-03 | -0.2576 | 0.0428 | * |
| -0.9~-0.6 vs 0.3~0.6 | 1.0794e-02 | 4.1572e-03 | 1.7550e-02 | 0.3328 | 0.0099 | ** |
| -0.9~-0.6 vs 0.6~0.9 | 1.6138e-02 | 1.0069e-02 | 2.2353e-02 | 0.5310 | 0.0000 | *** |
| -0.6~-0.3 vs -0.3~0 | 6.2024e-03 | -8.7624e-04 | 1.3157e-02 | 0.1847 | 0.1830 | n.s. |
| -0.6~-0.3 vs 0~0.3 | -9.7646e-03 | -1.6003e-02 | -3.4731e-03 | -0.3131 | 0.0186 | * |
| -0.6~-0.3 vs 0.3~0.6 | 8.6174e-03 | 1.5367e-03 | 1.5491e-02 | 0.2581 | 0.0457 | * |
| -0.6~-0.3 vs 0.6~0.9 | 1.3962e-02 | 7.1245e-03 | 2.0791e-02 | 0.4185 | 0.0030 | ** |
| -0.3~0 vs 0~0.3 | -1.5967e-02 | -2.2170e-02 | -9.6412e-03 | -0.5354 | 0.0000 | *** |
| -0.3~0 vs 0.3~0.6 | 2.4150e-03 | -4.4175e-03 | 9.2401e-03 | 0.0729 | 0.6338 | n.s. |
| -0.3~0 vs 0.6~0.9 | 7.7592e-03 | 1.4397e-03 | 1.3967e-02 | 0.2536 | 0.0457 | * |
| 0~0.3 vs 0.3~0.6 | 1.8382e-02 | 1.2264e-02 | 2.4364e-02 | 0.6323 | 0.0000 | *** |
| 0~0.3 vs 0.6~0.9 | 2.3726e-02 | 1.7721e-02 | 2.9676e-02 | 0.8171 | 0.0000 | *** |
| 0.3~0.6 vs 0.6~0.9 | 5.3442e-03 | -5.4232e-04 | 1.1494e-02 | 0.1767 | 0.2007 | n.s. |

CI denotes the 95% bootstrap confidence interval for the mean difference; the effect size (Cohen’s d) is based on paired samples; significance is based on FDR correction (Benjamini-Hochberg method): *** p < 0.001, ** p < 0.01, * p < 0.05, n.s. indicates not significant.

**Table. S3. Results of Pairwise Post-Hoc Comparisons for Each Time Window on D9.**

| D9 Comparison | MeanDiff | CI_lower | CI_upper | Cohen_d | p_FDR | Significance |
| --- | --- | --- | --- | --- | --- | --- |
| -0.9~-0.6 vs -0.6~-0.3 | -3.4462e-03 | -1.0742e-02 | 3.9433e-03 | -0.0977 | 0.5132 | n.s. |
| -0.9~-0.6 vs -0.3~0 | 1.5373e-04 | -7.8585e-03 | 8.0851e-03 | 0.0040 | 0.9691 | n.s. |
| -0.9~-0.6 vs 0~0.3 | -3.2641e-03 | -9.9103e-03 | 3.6414e-03 | -0.0978 | 0.5181 | n.s. |
| -0.9~-0.6 vs 0.3~0.6 | 1.0699e-02 | 3.2576e-03 | 1.7766e-02 | 0.3030 | 0.0228 | * |
| -0.9~-0.6 vs 0.6~0.9 | 1.3380e-02 | 6.0870e-03 | 2.0365e-02 | 0.3892 | 0.0024 | ** |
| -0.6~-0.3 vs -0.3~0 | 3.5999e-03 | -3.4291e-03 | 1.0687e-02 | 0.1049 | 0.4975 | n.s. |
| -0.6~-0.3 vs 0~0.3 | 1.8205e-04 | -6.1699e-03 | 6.8380e-03 | 0.0057 | 0.9653 | n.s. |
| -0.6~-0.3 vs 0.3~0.6 | 1.4146e-02 | 6.8144e-03 | 2.1455e-02 | 0.4089 | 0.0009 | *** |
| -0.6~-0.3 vs 0.6~0.9 | 1.6826e-02 | 9.4117e-03 | 2.4408e-02 | 0.4666 | 0.0000 | *** |
| -0.3~0 vs 0~0.3 | -3.4179e-03 | -1.0292e-02 | 3.3146e-03 | -0.1029 | 0.5024 | n.s. |
| -0.3~0 vs 0.3~0.6 | 1.0546e-02 | 3.1237e-03 | 1.7914e-02 | 0.2933 | 0.0255 | * |
| -0.3~0 vs 0.6~0.9 | 1.3226e-02 | 5.7050e-03 | 2.0747e-02 | 0.3610 | 0.0039 | ** |
| 0~0.3 vs 0.3~0.6 | 1.3964e-02 | 7.9170e-03 | 1.9862e-02 | 0.4914 | 0.0000 | *** |
| 0~0.3 vs 0.6~0.9 | 1.6644e-02 | 1.0448e-02 | 2.2714e-02 | 0.5556 | 0.0000 | *** |
| 0.3~0.6 vs 0.6~0.9 | 2.6803e-03 | -3.7701e-03 | 9.2023e-03 | 0.0850 | 0.5643 | n.s. |

CI denotes the 95% bootstrap confidence interval for the mean difference; the effect size (Cohen’s d) is based on paired samples; significance is based on FDR correction (Benjamini-Hochberg method): *** p < 0.001, ** p < 0.01, * p < 0.05, n.s. indicates not significant.

**Table. S4. Results of Pairwise Post-Hoc Comparisons for Each Time Window on A9.**

| A9 Comparison | MeanDiff | CI_lower | CI_upper | Cohen_d | p_FDR | Significance |
| --- | --- | --- | --- | --- | --- | --- |
| -0.9~-0.6 vs -0.6~-0.3 | 2.0537e-04 | -6.9275e-03 | 7.3838e-03 | 0.0060 | 0.9653 | n.s. |
| -0.9~-0.6 vs -0.3~0 | 3.1365e-03 | -3.9171e-03 | 1.0132e-02 | 0.0898 | 0.5478 | n.s. |
| -0.9~-0.6 vs 0~0.3 | -5.2848e-03 | -1.3499e-02 | 2.9155e-03 | -0.1325 | 0.3539 | n.s. |
| -0.9~-0.6 vs 0.3~0.6 | 1.0555e-02 | 3.8033e-03 | 1.7319e-02 | 0.3304 | 0.0137 | * |
| -0.9~-0.6 vs 0.6~0.9 | 2.4756e-03 | -5.6611e-03 | 1.0458e-02 | 0.0631 | 0.6867 | n.s. |
| -0.6~-0.3 vs -0.3~0 | 2.9311e-03 | -4.0004e-03 | 9.4853e-03 | 0.0898 | 0.5525 | n.s. |
| -0.6~-0.3 vs 0~0.3 | -5.4902e-03 | -1.2807e-02 | 1.6028e-03 | -0.1598 | 0.2548 | n.s. |
| -0.6~-0.3 vs 0.3~0.6 | 1.0350e-02 | 3.9981e-03 | 1.6756e-02 | 0.3303 | 0.0129 | * |
| -0.6~-0.3 vs 0.6~0.9 | 2.2702e-03 | -5.4928e-03 | 1.0186e-02 | 0.0593 | 0.6960 | n.s. |
| -0.3~0 vs 0~0.3 | -8.4213e-03 | -1.5195e-02 | -1.6914e-03 | -0.2621 | 0.0428 | * |
| -0.3~0 vs 0.3~0.6 | 7.4188e-03 | 1.1535e-03 | 1.3660e-02 | 0.2487 | 0.0503 | n.s. |
| -0.3~0 vs 0.6~0.9 | -6.6095e-04 | -7.5468e-03 | 6.3731e-03 | -0.0195 | 0.9089 | n.s. |
| 0~0.3 vs 0.3~0.6 | 1.5840e-02 | 9.5116e-03 | 2.2271e-02 | 0.5171 | 0.0000 | *** |
| 0~0.3 vs 0.6~0.9 | 7.7604e-03 | 5.7498e-04 | 1.4904e-02 | 0.2216 | 0.0904 | n.s. |
| 0.3~0.6 vs 0.6~0.9 | -8.0797e-03 | -1.4415e-02 | -1.9312e-03 | -0.2678 | 0.0374 | * |

CI denotes the 95% bootstrap confidence interval for the mean difference; the effect size (Cohen’s d) is based on paired samples; significance is based on FDR correction (Benjamini-Hochberg method): *** p < 0.001, ** p < 0.01, * p < 0.05, n.s. indicates not significant.

**Table. S5. Results of Pairwise Post-Hoc Comparisons for Each Time Window on F11.**

| F11 Comparison | MeanDiff | CI_lower | CI_upper | Cohen_d | p_FDR | Significance |
| --- | --- | --- | --- | --- | --- | --- |
| -0.9~-0.6 vs -0.6~-0.3 | 3.9268e-03 | -3.6187e-03 | 1.1124e-02 | 0.1120 | 0.4578 | n.s. |
| -0.9~-0.6 vs -0.3~0 | 2.1318e-03 | -6.5374e-03 | 1.1001e-02 | 0.0501 | 0.7575 | n.s. |
| -0.9~-0.6 vs 0~0.3 | -9.9085e-03 | -1.8659e-02 | -1.2443e-03 | -0.2351 | 0.0704 | n.s. |
| -0.9~-0.6 vs 0.3~0.6 | 8.9431e-03 | -2.6350e-04 | 1.8165e-02 | 0.1994 | 0.1451 | n.s. |
| -0.9~-0.6 vs 0.6~0.9 | 4.9603e-04 | -9.5127e-03 | 1.0521e-02 | 0.0103 | 0.9596 | n.s. |
| -0.6~-0.3 vs -0.3~0 | -1.7950e-03 | -1.0119e-02 | 6.0518e-03 | -0.0458 | 0.7675 | n.s. |
| -0.6~-0.3 vs 0~0.3 | -1.3835e-02 | -2.2020e-02 | -6.0335e-03 | -0.3611 | 0.0049 | ** |
| -0.6~-0.3 vs 0.3~0.6 | 5.0162e-03 | -3.2329e-03 | 1.3511e-02 | 0.1215 | 0.4070 | n.s. |
| -0.6~-0.3 vs 0.6~0.9 | -3.4308e-03 | -1.3084e-02 | 5.6791e-03 | -0.0775 | 0.6112 | n.s. |
| -0.3~0 vs 0~0.3 | -1.2040e-02 | -1.9828e-02 | -4.1296e-03 | -0.3196 | 0.0140 | * |
| -0.3~0 vs 0.3~0.6 | 6.8113e-03 | -1.3578e-03 | 1.5070e-02 | 0.1707 | 0.2157 | n.s. |
| -0.3~0 vs 0.6~0.9 | -1.6358e-03 | -1.0624e-02 | 7.6527e-03 | -0.0370 | 0.8054 | n.s. |
| 0~0.3 vs 0.3~0.6 | 1.8852e-02 | 1.1956e-02 | 2.5631e-02 | 0.5636 | 0.0000 | *** |
| 0~0.3 vs 0.6~0.9 | 1.0405e-02 | 3.1781e-03 | 1.7972e-02 | 0.2921 | 0.0202 | * |
| 0.3~0.6 vs 0.6~0.9 | -8.4470e-03 | -1.6046e-02 | -1.0164e-03 | -0.2316 | 0.0828 | n.s. |

CI denotes the 95% bootstrap confidence interval for the mean difference; the effect size (Cohen’s d) is based on paired samples; significance is based on FDR correction (Benjamini-Hochberg method): *** p < 0.001, ** p < 0.01, * p < 0.05, n.s. indicates not significant.

**Table. S6. Results of Pairwise Post-Hoc Comparisons for Each Time Window on B10.**

| B10 Comparison | MeanDiff | CI_lower | CI_upper | Cohen_d | p_FDR | Significance |
| --- | --- | --- | --- | --- | --- | --- |
| -0.9~-0.6 vs -0.6~-0.3 | 1.6880e-03 | -3.4795e-03 | 7.0613e-03 | 0.0665 | 0.6664 | n.s. |
| -0.9~-0.6 vs -0.3~0 | 4.1883e-04 | -4.8040e-03 | 5.5574e-03 | 0.0166 | 0.9236 | n.s. |
| -0.9~-0.6 vs 0~0.3 | -6.3641e-03 | -1.0781e-02 | -1.8103e-03 | -0.2914 | 0.0210 | * |
| -0.9~-0.6 vs 0.3~0.6 | -3.0831e-03 | -8.4993e-03 | 2.1458e-03 | -0.1194 | 0.4129 | n.s. |
| -0.9~-0.6 vs 0.6~0.9 | 1.5075e-03 | -3.9868e-03 | 7.0116e-03 | 0.0554 | 0.7207 | n.s. |
| -0.6~-0.3 vs -0.3~0 | -1.2692e-03 | -6.0975e-03 | 3.4006e-03 | -0.0540 | 0.7303 | n.s. |
| -0.6~-0.3 vs 0~0.3 | -8.0522e-03 | -1.3026e-02 | -3.0679e-03 | -0.3393 | 0.0110 | * |
| -0.6~-0.3 vs 0.3~0.6 | -4.7712e-03 | -9.9032e-03 | 1.8342e-04 | -0.1940 | 0.1451 | n.s. |
| -0.6~-0.3 vs 0.6~0.9 | -1.8055e-04 | -5.3551e-03 | 5.1943e-03 | -0.0071 | 0.9653 | n.s. |
| -0.3~0 vs 0~0.3 | -6.7829e-03 | -1.2087e-02 | -1.3432e-03 | -0.2610 | 0.0428 | * |
| -0.3~0 vs 0.3~0.6 | -3.5020e-03 | -8.2193e-03 | 1.3039e-03 | -0.1516 | 0.2730 | n.s. |
| -0.3~0 vs 0.6~0.9 | 1.0887e-03 | -4.2513e-03 | 6.4528e-03 | 0.0419 | 0.7860 | n.s. |
| 0~0.3 vs 0.3~0.6 | 3.2810e-03 | -1.6066e-03 | 8.1189e-03 | 0.1357 | 0.3480 | n.s. |
| 0~0.3 vs 0.6~0.9 | 7.8716e-03 | 3.0333e-03 | 1.2745e-02 | 0.3313 | 0.0115 | * |
| 0.3~0.6 vs 0.6~0.9 | 4.5906e-03 | -1.0886e-03 | 1.0476e-02 | 0.1624 | 0.2507 | n.s. |

CI denotes the 95% bootstrap confidence interval for the mean difference; the effect size (Cohen’s d) is based on paired samples; significance is based on FDR correction (Benjamini-Hochberg method): *** p < 0.001, ** p < 0.01, * p < 0.05, n.s. indicates not significant.

**Table. S7. Results of Pairwise Post-Hoc Comparisons for Each Time Window on G10.**

| G10 Comparison | MeanDiff | CI_lower | CI_upper | Cohen_d | p_FDR | Significance |
| --- | --- | --- | --- | --- | --- | --- |
| -0.9~-0.6 vs -0.6~-0.3 | -5.6414e-03 | -1.2825e-02 | 1.3376e-03 | -0.1638 | 0.2333 | n.s. |
| -0.9~-0.6 vs -0.3~0 | -5.1127e-03 | -1.2402e-02 | 2.4376e-03 | -0.1429 | 0.3138 | n.s. |
| -0.9~-0.6 vs 0~0.3 | -3.8350e-03 | -1.1839e-02 | 4.8454e-03 | -0.0952 | 0.5351 | n.s. |
| -0.9~-0.6 vs 0.3~0.6 | -1.3792e-03 | -9.5375e-03 | 7.4388e-03 | -0.0338 | 0.8193 | n.s. |
| -0.9~-0.6 vs 0.6~0.9 | 6.9168e-03 | -1.9444e-03 | 1.6402e-02 | 0.1558 | 0.2587 | n.s. |
| -0.6~-0.3 vs -0.3~0 | 5.2872e-04 | -6.3751e-03 | 7.6631e-03 | 0.0157 | 0.9236 | n.s. |
| -0.6~-0.3 vs 0~0.3 | 1.8064e-03 | -6.2320e-03 | 1.0304e-02 | 0.0453 | 0.7675 | n.s. |
| -0.6~-0.3 vs 0.3~0.6 | 4.2623e-03 | -5.2750e-03 | 1.3806e-02 | 0.0924 | 0.5478 | n.s. |
| -0.6~-0.3 vs 0.6~0.9 | 1.2558e-02 | 2.7340e-03 | 2.1577e-02 | 0.2743 | 0.0356 | * |
| -0.3~0 vs 0~0.3 | 1.2777e-03 | -6.2875e-03 | 9.1262e-03 | 0.0347 | 0.8171 | n.s. |
| -0.3~0 vs 0.3~0.6 | 3.7336e-03 | -5.1665e-03 | 1.2118e-02 | 0.0884 | 0.5478 | n.s. |
| -0.3~0 vs 0.6~0.9 | 1.2030e-02 | 2.6699e-03 | 2.1679e-02 | 0.2651 | 0.0428 | * |
| 0~0.3 vs 0.3~0.6 | 2.4558e-03 | -5.0036e-03 | 9.9065e-03 | 0.0684 | 0.6536 | n.s. |
| 0~0.3 vs 0.6~0.9 | 1.0752e-02 | 2.6447e-03 | 1.8996e-02 | 0.2711 | 0.0398 | * |
| 0.3~0.6 vs 0.6~0.9 | 8.2960e-03 | -8.0625e-04 | 1.7010e-02 | 0.1918 | 0.1525 | n.s. |

CI denotes the 95% bootstrap confidence interval for the mean difference; the effect size (Cohen’s d) is based on paired samples; significance is based on FDR correction (Benjamini-Hochberg method): *** p < 0.001, ** p < 0.01, * p < 0.05, n.s. indicates not significant.

**Table. S8. Results of Pairwise Post-Hoc Comparisons for Each Time Window on E10.**

| E10 Comparison | MeanDiff | CI_lower | CI_upper | Cohen_d | p_FDR | Significance |
| --- | --- | --- | --- | --- | --- | --- |
| -0.9~-0.6 vs -0.6~-0.3 | -9.7792e-03 | -1.7443e-02 | -1.8703e-03 | -0.2606 | 0.0428 | * |
| -0.9~-0.6 vs -0.3~0 | -5.3777e-03 | -1.2601e-02 | 2.0475e-03 | -0.1527 | 0.2730 | n.s. |
| -0.9~-0.6 vs 0~0.3 | -6.5885e-03 | -1.5501e-02 | 2.2587e-03 | -0.1541 | 0.2637 | n.s. |
| -0.9~-0.6 vs 0.3~0.6 | 4.1946e-03 | -4.0157e-03 | 1.2434e-02 | 0.1050 | 0.4915 | n.s. |
| -0.9~-0.6 vs 0.6~0.9 | 1.0265e-02 | 3.3689e-04 | 2.0294e-02 | 0.2119 | 0.1095 | n.s. |
| -0.6~-0.3 vs -0.3~0 | 4.4015e-03 | -2.9383e-03 | 1.1775e-02 | 0.1240 | 0.4027 | n.s. |
| -0.6~-0.3 vs 0~0.3 | 3.1907e-03 | -5.8614e-03 | 1.1988e-02 | 0.0740 | 0.6338 | n.s. |
| -0.6~-0.3 vs 0.3~0.6 | 1.3974e-02 | 5.2902e-03 | 2.2901e-02 | 0.3257 | 0.0110 | * |
| -0.6~-0.3 vs 0.6~0.9 | 2.0044e-02 | 1.0817e-02 | 2.9408e-02 | 0.4462 | 0.0009 | *** |
| -0.3~0 vs 0~0.3 | -1.2108e-03 | -7.7777e-03 | 5.2404e-03 | -0.0383 | 0.8054 | n.s. |
| -0.3~0 vs 0.3~0.6 | 9.5723e-03 | 1.7867e-03 | 1.7231e-02 | 0.2596 | 0.0438 | * |
| -0.3~0 vs 0.6~0.9 | 1.5643e-02 | 7.0824e-03 | 2.3757e-02 | 0.3830 | 0.0035 | ** |
| 0~0.3 vs 0.3~0.6 | 1.0783e-02 | 3.8980e-03 | 1.7848e-02 | 0.3198 | 0.0147 | * |
| 0~0.3 vs 0.6~0.9 | 1.6853e-02 | 9.0543e-03 | 2.4508e-02 | 0.4463 | 0.0000 | *** |
| 0.3~0.6 vs 0.6~0.9 | 6.0702e-03 | -1.8936e-03 | 1.3626e-02 | 0.1590 | 0.2587 | n.s. |

CI denotes the 95% bootstrap confidence interval for the mean difference; the effect size (Cohen’s d) is based on paired samples; significance is based on FDR correction (Benjamini-Hochberg method): *** p < 0.001, ** p < 0.01, * p < 0.05, n.s. indicates not significant.

**Table. S9 The rate of change in TE between the task period and the daily state.**

| Time | A9 | B10 | C10 | D9 | E10 | F11 | G10 | H9 |
| --- | --- | --- | --- | --- | --- | --- | --- | --- |
| -0.9~-0.6 | 0.1461 |  | 0.1156 | 0.1575 | 0.1708 | 0.1120 | 0.1430 | 0.1930 |
| -0.6~-0.3 | 0.1058 |  | 0.0695 | 0.2924 | 0.2810 |  | 0.2188 | 0.1377 |
| -0.3~0 | 0.0692 |  | 0.0840 | 0.2148 | 0.2224 |  | 0.2119 |  |
| 0~0.3 | 0.2018 | 0.1344 | 0.1652 | 0.2165 | 0.2340 | 0.2497 | 0.2164 | 0.3406 |
| 0.3~0.6 |  |  | 0.1797 |  |  |  | 0.1984 |  |
| 0.6~0.9 | 0.1421 |  | 0.1661 |  |  | 0.1493 |  |  |

**Table. S10 Description of the statistical methods corresponding to the figures**

| Figure number | Biological No. | Total Trials / Factors | Outcome | Statistical Testing Methods |
| --- | --- | --- | --- | --- |
| Fig.4b | 1（Pt. Y） | 2*8*89*10 (Task/rest * Channels * trials * time windows) | Detrended intercept (β₀) from regression model applied to the paired ln(MS_Task) – ln(MS_Rest) difference | Two one-sided t-tests (TOST-equivalent) on β₀ vs. 0, per channel & window; P-values (2×8×10=160 tests) adjusted via Benjamini-Hochberg FDR (q<0.05). |
| Fig.5d/Table.S1-S8 | 1（Pt. Y） | 8*89*6 (Channels * trials * time windows) | Transfer Entropy (TE) values across 89 trials each 300-ms time-window (-0.9 to 0.9 s). | Main effect: One-way repeated-measures permutation ANOVA (10,000 label permutations, shuffling time-window labels within each trial) on TE values. Post-hoc: Paired permutation t-tests (10,000 permutations) on significant channels/pairs across 15 pairwise time-window comparisons, with FDR correction applied to all post-hoc p-values. |
| Fig.7e, f | 4 | 19 total seizure events |  | **e.** Paired t-test, *P < 0.05. **f**. one-sample t-test, *P < 0.05. |

CI denotes the 95% bootstrap confidence interval for the mean difference; the effect size (Cohen’s d) is based on paired samples; significance is based on FDR correction (Benjamini-Hochberg method): *** p < 0.001, ** p < 0.01, * p < 0.05, n.s. indicates not significant.

**Supplementary Materials and Methods**

**Animals and housing**

Male C57BL/6 mice aged 8 weeks and male Sprague-Dawley rats aged 6–8 weeks and weighing 300–350 g at the time of the experiments were used. Animals were housed in standard cages under a 12-h light/12-h dark cycle, with ad libitum access to food and water. Health status was monitored daily. After surgery, animals received three days of postoperative care, including wound management and analgesia. All animal experiments were conducted in accordance with the Chinese guideline for ethical review of laboratory animal welfare (GB/T 35892-2018) and approved by the Institutional Animal Care and Use Committee of Nanjing Medical University (IACUC No. 2507026 for mouse experiments and IACUC No. 2026002 for rat experiments ).

**Induction of seizure onsets with chemogenetic activation of hippocampal excitatory neurons in mice**

This chemogenetic mouse experiment included four mice and generated 19 seizure events for the EEG and astrocytic Ca²⁺ timing analysis. Mice were anesthetized with 2% isoflurane and placed in a stereotaxic apparatus. AAV2/9-mCaMKIIα-hM3D(Gq)-mCherry (70 nL) was bilaterally injected into the hippocampus (AP, −2.5 mm; ML, ±3.2 mm; DV, −2.4 mm). EEG and EMG electrodes were implanted during the same surgery. After a three-week recovery period, clozapine-N-oxide (CNO; 0.5 mg/kg) was administered intraperitoneally to induce seizure activity. No animals were excluded from this 4-mouse chemogenetic cohort; all 4 mice completed full recording sessions and contributed to the 19 analyzed seizure events.

**Fiber photometry for measuring calcium activity in astrocytes in mice**

For the same four-mouse chemogenetic cohort, AAV2/5-GFAP-GCaMP6s-EGFP-WPRE-pA (1 × 10¹³ vg/mL; 200 nL) was infused into the unilateral hippocampus together with the hM3D(Gq) construct. An optic fiber was implanted at the injection site (AP: -2.5 mm; ML, +3.2 mm; DV, −2.1 mm).

**EEG and EMG recording and analysis in mice**

For the same four-mouse chemogenetic cohort, EEG electrodes were implanted over the frontal and parietal cortices, and two EMG wires were inserted into the trapezius muscles. Conventional EEG and EMG signals were analyzed at 0.5–30 Hz and 20–200 Hz, respectively, using SleepSign software (Kissei Comtec, Nagano, Japan). These settings apply to the conventional EEG/photometry experiment that generated the 19 events from four mice and do not describe the acquisition bandwidth of the separate mouse Sv DC field-potential experiment. The results involving this part of the data are presented in Fig. 7c–f.

**Fiber photometry *in vivo* recording of hippocampal astrocytic Ca^2+^ signaling in mice**

For photometry recordings in the same chemogenetic cohort of four mice, approximately 200 nL of AAV2/5-GFAP-GCaMP6s-EGFP-WPRE-pA (1 × 10¹³ vg/mL) was injected into the unilateral hippocampus, and an optic fiber was implanted at the injection site (AP, -2.5 mm; ML, ±3.2 mm; DV, −2.1 mm). A fiber-optic cannula, optic-fiber jumper, and ceramic sleeve (FOC-C-W-1.25-200-0.37-2.4; Hangzhou Sanshi Biotechnology) were connected to a commercial fiber-photometry system (Thinker Tech, Nanjing, China). Analog photometry signals were digitized at 512 Hz using a Power 1401 digitizer and Spike2 software (CED, Cambridge, UK). Fluorescence signals were converted to ΔF/F using ΔF/F = (F − F₀)/F₀, where F₀ was the mean baseline fluorescence.

**Fig. S9 Flowchart of epilepsy induction and data collection in mice**

**Surgical procedures for electrophysiology in mice**

Field-potential signals were acquired using a Neurostudio recording system (Changxiang Medical Limited) at a sampling rate of 30 kHz. The original acquisition files retained the 0.01–0.5 Hz components used for the mouse Sv DC analysis. These recordings were separate from the conventional EEG and astrocytic Ca²⁺ photometry experiment described above. The results involving this part of the data are presented in Fig. 7a,b.

**Fig. S10 Flowchart of epilepsy induction and data collection in mice**

**Surgical procedures for electrophysiology in rats**

This rat electrophysiological experiment included two rats. No animals were excluded after initial enrollment; both rats completed the full surgical and recording procedures and were fully included in the final statistical analysis. Rats were anesthetized by induction with 5% isoflurane. The fur was shaved and the skin was disinfected. Atropine (1 mg/kg body weight) and carprofen (5 mg/kg body weight) were administered intraperitoneally. The animals were placed in a stereotaxic apparatus, and anesthesia was maintained with 1–1.5% isoflurane during surgery.

After scalp incision, 18 cranial openings were prepared according to the ECoG probe coordinates. A 3 mm × 3 mm bone window was drilled over the left hippocampus, and the ECoG probe was fixed to the skull using microscrews. HC-030031 or vehicle control was administered intraperitoneally before recording, with recording initiated after the stated drug-exposure interval. A volume of 300 nL 4-aminopyridine was delivered to hippocampal CA1 using a nano-injector (reported coordinates: AP, −4.6 mm; ML, ±2.0 mm; DV, −3.5 mm). A 16-channel microelectrode array was then implanted into the hippocampus, and ground and reference wires were fixed near the cerebellum with cranial screws. The results involving this part of the data are presented in Fig. S7.

**Fig. S11 Flowchart of epilepsy induction and data collection in SD rats**

**Neural signal acquisition and analysis in rats**

Neural signals were recorded from the two rats under light anesthesia maintained with 1% isoflurane. Rat A initially received a control injection (vehicle), with neural data collected from 45 to 105 minutes post-injection. Subsequently, a TRPA1 inhibitor was administered, and data were acquired between 30 and 100 minutes post-inhibitor injection. Rat B received only the inhibitor, with data collected during the identical 30–100 minute post-injection window. ECoG signals (channels 17–32) and hippocampal local field-potential signals (channels 1–16) were recorded using a Neurostudio system at 30 kHz. A 50 Hz notch filter was applied for power-line interference. The original acquisition files contained the 0.01–0.5 Hz components used for low-frequency analysis.

A custom MATLAB program parsed the native .btn files and extracted signal traces and channel configurations. Signals were converted to .csv format and downsampled to 1000 Hz. All subsequent filtering operations were implemented as bidirectional zero-phase procedures to eliminate phase distortion, a critical requirement for preserving the temporal dynamics of the ultra-slow Sv DC components. Digital notch filtering was applied at 50, 100, 150, 200, and 250 Hz, followed by a fourth-order zero-phase Butterworth band-pass filter at 0.01–249 Hz. A subsequent digital fourth-order zero-phase Butterworth 0.01–0.5 Hz band-pass filter was used to isolate the low-frequency component for Sv DC feature extraction.

**Statistical analysis in rats**

ECoG segments were extracted separately for each of the two rats after HC-030031 or vehicle administration, focusing on seizure-associated periods. Three unstable channels (Channel 17 and Channel 18) were excluded according to the source analysis. Signal dispersion was quantified as the time-averaged variance across retained channels. RMS was calculated as the amplitude-related metric for each individual ECoG channel from the isolated 0.01–0.5 Hz Sv DC traces, producing a value inherently independent of the total duration of the analyzed seizure segment.The Wilcoxon rank-sum test was used for within-animal exploratory comparisons of feature values between early and late recording phases under the same injection condition (vehicle or HC-030031). All test results are interpreted strictly as descriptive within-animal observations.

**Order and Performance Testing of Filters**

Since this study involves ultra-low-frequency filtering of SEEG signals, synthetic-signal recovery tests were conducted to evaluate the impact of the filter order and filtering distortion at the beginning and end of the data to ensure that the filter parameters were set appropriately to capture signals in the Sv DC band. First, we generated a 0.3 Hz sine wave with a duration of 1000 s and superimposed a 1-second-wide short-duration pulse at both the beginning (1–2 s) and the end (998–999 s). We then applied 0.01–0.5 bandpass zero-phase filtering using Butter filters of different orders. The short pulses added at the beginning and end represent an extreme case; normally, signals do not exhibit such high levels of noise at their ends. Under these extreme conditions, both 2nd- and 4th-order Butterworth filters were able to limit the decay oscillation time at the beginning and end to within 60 s. However, when the filter order is higher, prolonged decay distortion does indeed occur at both ends of the signal. Specifically, a 6th-order Butterworth filter resulted in approximately 300 s of distortion at the beginning and end, while an 8th-order Butterworth filter resulted in approximately 400 s. This study employed a 4th-order Butterworth filter, retaining complete data sets at both the beginning and end (with approximately 10 minutes of data collected before and after the task), which effectively minimized the impact of filter distortion on data collected during the task. If higher-order filters are required, care must be taken to account for filtering distortion at the beginning and end of the data.

Fig. S12 The filtering performance of filters of different orders at the beginning and end of a signal during ultra-low-frequency bandpass filtering. Figures a–d show the results for filters of orders 2, 4, 6, and 8, respectively, where the blue lines represent the sine wave signals with interference at the beginning and end of the band, and the red curves represent the filtering results.

We processed real SEEG data from Pt. Y using four-order filters in the 0.01–0.5 Hz range. The data set used comprised the complete recording from start to finish (1,580 seconds in total), including 613 seconds of resting state before the task, 356 seconds during the task, and 611 seconds of post-task recording. The H9 channel was extracted for bandpass filtering, and the segments from 149 to 177 seconds (resting state) and 649 to 677 seconds (visual task) were selected to demonstrate the filtering results. Since real SEEG data does not contain the extreme waveform transients intentionally introduced in the simulation above, the filtering results for filters of orders 4, 6, and 8 on the task-state signal are nearly identical, and all provide a good fit to the low-frequency waveforms of the original signal. Because the filter of order 8 has a longer decay time at the beginning, it introduces oscillatory distortion when processing the resting-state data (149–177 s) near the start of the complete dataset.

Fig. S13 Filtering results for filters of different orders applied to real SEEG signals. Figures a–d show the results for filters of orders 2, 4, 6, and 8, respectively, where the blue line represents the raw SEEG signal and the red curve represents the filtered result. The left side of each subfigure shows 28 seconds of data collected during the resting state, while the right side shows 28 seconds of SEEG data collected during the task state.
